# When the body resonates with the pain of the other: Empathy Bodyssence in Parkinson’s disease

**DOI:** 10.1093/nc/niag010

**Published:** 2026-04-07

**Authors:** María del Carmen Tejada, Antonia Zepeda, Alejandro Troncoso, Anaís Aluicio, Rebecca M Todd, David Martínez-Pernía

**Affiliations:** Center for Social and Cognitive Neuroscience (CSCN), School of Psychology, Universidad Adolfo Ibanez, Santiago, Chile; Escuela de Psicologia, Universidad del Alba, Santiago, Chile; Center for Social and Cognitive Neuroscience (CSCN), School of Psychology, Universidad Adolfo Ibanez, Santiago, Chile; School of Kinesiology, Faculty of Rehabilitation Sciences and Quality of Life, Universidad San Sebastián, Valdivia 5090000, Chile; Center for Social and Cognitive Neuroscience (CSCN), School of Psychology, Universidad Adolfo Ibanez, Santiago, Chile; Department of Psychology, The University of British Columbia, Vancouver, British Columbia, Canada; Center for Social and Cognitive Neuroscience (CSCN), School of Psychology, Universidad Adolfo Ibanez, Santiago, Chile; Geroscience Center for Health and Brain Metabolism (GERO), Santiago, Chile

**Keywords:** empathy, parkinson’s disease, enaction, phenomenology, neurophenomenology, Bodyssence

## Abstract

Empathy plays a fundamental role in social bonding and intersubjective understanding. While recent research has emphasized how bodily processes shape empathic engagement and underlie individual differences, the impact of bodily disruptions on empathic experience in Parkinson’s disease (PD) remains largely unexplored. In this study, we used a neurophenomenological approach to investigate Empathy Bodyssence in PD, conceived as an enacted organization of bodily, affective, and meaning-related dimensions of experience, by integrating first-person experiential data with motor, physiological, and self-report measures. Forty-two individuals with PD watched pain-related and baseline videos. Empathic engagement was assessed through self-reports, postural movement, and physiological recordings (heart rate and electrodermal activity). Following exposure, participants underwent phenomenological interviews designed to capture their experience of witnessing another’s suffering. The interview data were analyzed through an iterative, multistage process involving independent coding, triangulation, and advanced analytical tools (CAQDAS, inter-rater agreement assessment, and interactive dashboards) to ensure analytical depth and rigor. Phenomenological analysis enabled us to categorize participants into two groups, distinguished by high versus low levels of bodily resonance in response to viewing pain. These phenomenological groupings were then integrated with quantitative data to reveal two distinct structures through which individuals with PD embodied empathy: Resonance Bodyssence, a response in which emotions are tightly coupled with bodily sensations and movement; and Marginal Resonance Bodyssence, a more observational and cognitively mediated response, marked by reduced bodily resonance. By using phenomenological structure as an organizing level of analysis, the present study shows how interindividual variability in motor and physiological responses in PD is enacted as distinct embodied modes of empathic engagement. In doing so, it advances neurophenomenological approaches and provides a more nuanced, embodied account of empathy in PD as a heterogeneous and dynamically enacted phenomenon.

## Highlights

- A neurophenomenological approach integrates lived experience with motor and physiological dynamics to study empathy in Parkinson’s disease.
- Bodyssence captures how bodily, affective, and meaning-related dimensions co-emerge as distinct empathic structures in Parkinson’s disease.
- Resonance Bodyssence reflects a coordinated coupling of bodily sensations, emotional activation, and sensorimotor engagement during empathic experience.
- Marginal Resonance Bodyssence reflects a mode of empathy dominated by observational processing, with limited bodily integration and reduced motor–affective coupling.
- Phenomenological organization renders interindividual motor and physiological variability intelligible.

## Introduction

Empathy is a capacity that is fundamental for sustaining human social life, supporting cooperation, mutual support, and conflict resolution (Shamay-Tsoory et al. 2004; Beadle and de la Vega 2019). Defined as the ability to recognize, feel, and share the experiences of others (Håkansson Eklund and Summer Meranius 2021), empathy is increasingly understood to be grounded in bodily processes such as sensorimotor dynamics and autonomic responses (Colombetti 2014; Troncoso et al. 2024; Monnier et al. 2025). This bodily dimension is particularly relevant for affective empathy, as—unlike other forms of social cognition that rely predominantly on inferential or evaluative processes—empathy for another’s suffering entails an immediate bodily involvement that is expressed behaviorally through sensorimotor resonance, autonomic reactivity, and action-oriented dispositions toward others (Decety and Jackson 2004; DiGirolamo et al. 2019; Plata-Bello et al. 2023). Although cognitive aspects of social cognition in Parkinson’s disease (PD) have been extensively studied (Coundouris et al. 2020; Dodich et al. 2022), the role of bodily disruptions in shaping empathic experience remains largely unexplored. To address this gap, we adopt a refined and embodied neurophenomenological approach inspired by Varela’s original program (Varela 1996), integrating first-person phenomenological accounts with third-person neurophysiological measures (here used broadly to include autonomic and motor responses), as well as self-report data, to characterize distinct patterns of empathic engagement in PD. Here, ‘refined neurophenomenology’ refers to later developments of the program that explicitly extend beyond brain-centered measures to include bodily, physiological, and interactive dimensions of lived experience, as articulated in the Empirical 5E (E5E) approach (Troncoso et al. 2023).

Although empathy involves bodily, cognitive, and affective dimensions (Decety and Jackson 2004; Decety 2007), focusing exclusively on objective measures such as physiological or motor indices is insufficient. Empathy cannot be reduced to bodily activation since it also entails a subjective dimension where meaning and lived experience are constituted (Monnier et al. 2025; Troncoso et al. 2024; Olivares et al. 2015; Neumann and Westbury 2011; Gallagher and Zahavi, 2020). Addressing this conceptual and methodological challenge requires multilevel approaches that integrate physiological, behavioral, and subjective measures, offering a more comprehensive account of the phenomenon (Vieten et al. 2024).

In this context, phenomenology offers a compelling path forward by explicitly incorporating the first-person perspective of the lived body (Fuchs and Koch 2014). It underscores the body as the primary medium for experiencing and expressing empathy, revealing how bodily sensations, kinesthetic motivations, and emotions are inextricably linked to our capacity to resonate with others (Thompson 2007; Fuchs 2017; Cea and Martínez-Pernía 2023). Building on this perspective, our group has conducted empirical studies using experimental phenomenological methods that integrate first-person phenomenological analysis with quantitative measures (Martínez-Pernía 2022). For example, Martínez-Pernía et al. (2023) showed how participants’ kinesthetic impulses and bodily resonances gave rise to two divergent ways of empathy—or, in phenomenological terms, *two distinct experiential structures*: a self-centered empathy, marked by tension and withdrawal into one’s own discomfort, and an other-centered empathy, where attention expanded toward the suffering person and motivated bodily tendencies to approach and offer support. Likewise, Troncoso et al. (2025) revealed that phenomenological clustering in interactive contexts yielded four distinct ways of navigating empathic distress and compassion—ranging from relational disengagement to balanced compassionate support. These graded experiences were positively correlated with the ‘empathic concern’ trait assessed by the Interpersonal Reactivity Index (IRI). Most recently, Zepeda et al. (2025) applied this approach to PD, identifying a spectrum of embodied intersubjective synchrony that differentiated fully embodied from externally oriented empathic responses, which were associated with significant quantitative differences in how participants lived the other’s suffering.

Within this line of work, Empathy Bodyssence was introduced as a unifying framework to articulate converging phenomenological and embodied findings within an enactive approach to empathy. From this perspective, empathic experience is understood as an embodied, affective, and intersubjective process of sense-making enacted through the dynamic coupling between the lived body and its environment (Varela et al. 1991; Thompson 2007; Colombetti 2014; Troncoso et al. 2023). Bodyssence refers to the manner in which empathy is lived, capturing how bodily sensations, affective modulation, and cognitive orientations unfold together in the subject’s engagement with others. Rather than naming a discrete process or component, Bodyssence describes the co-constitution of bodily, affective, and sense-making dimensions through which empathic understanding is enacted in intersubjective contexts (Troncoso et al. 2024). Empirical support for this conceptualization was provided by Troncoso et al. (2024), who showed that distinct configurations of Empathy Bodyssence were associated with phenomenological structure, cardiac dynamics, and postural modulation during empathy for pain. In that study, variations in how bodily sensations and affective engagement were lived co-occurred with differentiated patterns of physiological and motor activity, supporting the idea that empathic experience is enacted as an integrated embodied configuration rather than as the sum of independent components.

Collectively, these studies illustrate how phenomenological analysis uncovers coherent experiential structures within heterogeneity and maps them onto distinct neurophysiological and self-report patterns. Crucially, phenomenological clustering carved coherence out of heterogeneity, revealing significant differences in how participants live the other’s suffering. This demonstrates how phenomenology not only describes but also organizes heterogeneous experiences into meaningful structures that can be linked with quantitative patterns.

Despite the success of this approach in previous research, its application to clinical populations remains largely unexplored. Understanding empathy from an embodied perspective is particularly relevant in neurodegenerative diseases, where motor, cognitive, and emotional impairments disrupt the ability to perceive, process, and express empathic experiences (Eddy and Cook 2018). PD presents a unique case for investigating the relationship between phenomenological experience and neurophysiology in empathy. Beyond its well-documented motor impairments, PD profoundly alters social cognition, interoception, and affective resonance (e.g. García-Cordero et al. 2016; Argaud et al. 2018; Alonso-Recio et al. 2021). Individuals with PD exhibit reduced autonomic responses, diminished facial expressivity (hypomimia), and altered bodily feedback, all of which are essential for embodied empathy (Ricciardi et al. 2017; Clark et al. 2019). Additionally, the dopaminergic alterations characteristic of PD are thought to disrupt action–perception coordination and embodied forms of social attunement, which may in turn influence how empathic experience is configured across individuals (Baez et al. 2016; Péron et al. 2017).

At the same time, PD is marked by a highly heterogeneous clinical progression, meaning that symptoms do not emerge, evolve, or impact patients uniformly over time. Individuals with PD differ markedly in the type, severity, and temporal course of motor, autonomic, affective, and cognitive symptoms, as well as in how these symptoms interact in daily life (Lewis et al. 2005; Wüllner et al. 2023). As a result, two patients at similar disease stages may present substantially different patterns of bodily regulation, action–perception coupling, and emotional responsivity. This variability suggests that different individuals may exhibit distinct neurophysiological and phenomenological patterns of empathy, reinforcing the need for methodologies capable of capturing the diverse ways in which PD affects embodied intersubjectivity. Given this heterogeneity, integrating phenomenological and neurophysiological approaches offers a crucial methodological advantage by organizing interindividual variability rather than averaging it out. If empathy is fundamentally embodied, then differences in bodily and physiological regulation in PD should be reflected in distinct lived experiences of empathic engagement, and *vice versa*. A neurophenomenological approach is therefore particularly well suited to investigate how heterogeneous disease trajectories give rise to differentiated experiential structures of empathy in PD.

To address this gap, the primary aim of this study is to investigate how the phenomenological experience of empathy for pain may be associated with specific patterns of self-report, motor, and physiological response in PD. Specifically, we extend the framework of Empathy Bodyssence to PD, examining how experiential structures align with neurophysiological responses to provide a more holistic and comprehensive account. For this purpose, this investigation integrated motor and physiological data using a mobile body dynamics recording system, combined with first-person phenomenological reports. Participants were exposed to videos depicting accidental physical harm (empathy-for-pain condition) and neutral videos (baseline condition). During exposure, postural movement, electrocardiographic, and electrodermal responses were recorded to capture behavioral and physiological modulations associated with empathic experience. Following this, an interview inspired by the microphenomenological interview (Petitmengin 2006) examined the multilayered dimensions of empathy for pain, including bodily sensations, emotions, thoughts, and motivational aspects. The interview material was analyzed using a recursive and multistep method that included independent coding, cross-validation among researchers, and the use of analytical tools such as CAQDAS software, inter-rater agreement assessment, and dynamic visualizations—ensuring depth, consistency, and methodological rigor in the analysis. While the phenomenological dimension of empathic experience in this sample has been previously reported (Zepeda et al. 2025), the present study advances this work by integrating first-person phenomenological descriptions with self-reports, motor, and physiological measures. This integration enables a neurophenomenological investigation of embodied empathy in PD grounded in converging first- and third-person data. Based on the previously identified experiential structures, we formulated the hypothesis that variations in the phenomenological depth of empathic engagement would be systematically associated with distinct patterns of affective, motor, and physiological responses. Specifically, we hypothesized that Embodied Resonance Empathy would be characterized by increased autonomic and postural reactivity, together with higher self-reported emotional activation, whereas Marginal Embodied Resonance Empathy would be associated with attenuated physiological and motor responses and lower emotional activation.

## Methods

### Participants

In this study, 42 individuals with a confirmed diagnosis of PD participated, in accordance with the criteria established by the Brain Bank of the Parkinson’s Disease Society of the United Kingdom (Hughes et al. 1992). Participant selection was conducted in collaboration with neurologists from Hospital del Salvador (Santiago, Chile), who were responsible for inviting them to the study and ensuring adherence to the inclusion and exclusion criteria. The inclusion criteria were age ≥60 years (mean age = 70.36 years, SD = 6.34) and a diagnosis of PD at a mild-to-moderate stage (Hoehn and Yahr stages 1.0–3.0) (Hoehn and Yahr 1998). This criterion ensured that participants could stand safely and independently during the experimental protocol while reducing the likelihood that severe motor/postural instability would confound center-of-pressure (COP)–based measures. Disease stage was determined by the treating neurologists as part of routine clinical assessment. Additional criteria included normal or corrected-to-normal vision and hearing. The exclusion criteria included sensory impairments that could interfere with the study’s procedures, as well diagnosis of cognitive impairment, defined as a Mini-Mental State Examination score of < 21 (Molina-Donoso et al. 2023). To characterize the sample, sociodemographic information including age, gender, educational level, and cognitive assessment (Mini-Mental State Examination, MMSE) was collected. This study was conducted in accordance with the ethical standards of the Declaration of Helsinki and received approval from the Scientific Ethics Committee of Servicio de Salud Metropolitano Oriente and the Ethics Committee for Research on Human Subjects at the Faculty of Medicine, University of Chile.

### Screening questionnaires

In this study, we administered the Spanish-validated versions of various neuropsychological, mood, and social cognition assessments. These instruments, which have undergone extensive validation for Spanish-speaking populations, offer reliable and culturally relevant measures to assess the participants’ psychological profiles.

Cognitive screening was conducted using the MMSE. The MMSE is a widely used tool for assessing global cognitive function, evaluating domains such as orientation, attention, memory, language, and visuospatial skills (Beaman et al. 2004). The test consists of 30 items, with a total possible score of 30 points, where higher scores indicate better cognitive function.

Mood was assessed using the Geriatric Depression Scale (GDS-15, short version) and the Generalized Anxiety Disorder Assessment (GAD-7). The GDS-15 is a self-report instrument specifically developed to screen for depressive symptoms in older adults, providing a reliable measure of emotional well-being. It yields a score ranging from 0 to 15, with higher scores indicating greater depressive symptomatology (Hoyl et al. 2000). The GAD-7 is a standardized tool designed to evaluate the severity of generalized anxiety symptoms, assessing key domains such as excessive worry, restlessness, and tension. Its total score ranges from 0 to 21, with higher scores reflecting more severe anxiety symptoms (García-Campayo et al. 2010).

Social cognition was evaluated using the IRI and the Mini-Social Cognition & Emotional Assessment (Mini-SEA). The IRI is a multidimensional measure of empathy that assesses both cognitive and affective aspects of interpersonal sensitivity, with higher scores indicating greater empathic abilities (Fernández et al. 2011) The Mini-SEA is a brief but comprehensive tool designed to evaluate social cognition and emotional processing, particularly in populations with neurodegenerative conditions. It assesses emotion recognition and theory of mind abilities, providing a total score ranging from 0 to 30, with higher scores indicating better social cognition performance (Bertoux et al. 2012).

### Emotional stimuli: construction and validation

To create the empathy-for-pain and baseline video conditions, we compiled 12 audiovisual scenes for each condition using materials available online under Creative Commons licenses. Each clip lasted on average between 7 and 11 s. The pain condition featured sportspersons undergoing severe physical accidents while engaging in extreme sports (e.g. parkour, high-altitude slacklining, acrobatic snowboarding). Scenes involving dismemberment, disfigurement, or death were excluded, as were stimuli with significant camera motion, shaking, or those likely to induce saccadic eye movements. By contrast, the baseline condition consisted of non-emotional domestic scenes involving naturalistic movement without bodily harm or affectively salient events. This condition controlled for visual motion and general sensorimotor engagement while minimizing emotional and empathic content, allowing contrasts with the pain condition to primarily reflect affective–empathic processing. The emotional properties of the stimuli were validated in a separate sample of 65 healthy young adults using the Self-Assessment Manikin (SAM) to assess valence, arousal, and dominance. Paired *t*-tests showed that the videos selected for the pain condition were assigned significantly lower valence (pain: mean = 3.77 ± 1.94; baseline: mean = 4.97 ± 2.39; *t*(64) = −7.24, *P* < .001), higher arousal (pain: mean = 6.40 ± 1.78; baseline: mean = 3.02 ± 2.24; *t*(64) = 24.91, *P* < .001), and lower dominance (pain: mean = 5.31 ± 2.68; baseline: mean = 7.66 ± 2.25; *t*(64) = −14.59, *P* < .001) than baseline stimuli. Notably, the same set of pain-related and baseline video stimuli has been previously applied in studies with older adult populations, showing comparable affective differentiation between conditions (Slachevsky et al. 2020; Pizarro et al. 2023). Finally, two 60-s videos were constructed (pain and baseline conditions), each containing seven scenes.

### Procedure

Participants took part in all experimental procedures at the Hospital del Salvador (Santiago, Chile). After referral by the neurologist to the study, a psychologist supervised the informed consent process and verified that each participant met the inclusion criteria through a brief interview and cognitive screening. The psychologist also administered social cognition and mood scales. To control for potential effects of PD symptoms and to reduce motor symptom expression during the experimental protocol, all participants were assessed in the ON medication state. In accordance with medication timing protocols reported in previous studies and the recommendations of the treating neurologists (Tykalova et al. 2022), participants took their prescribed dopaminergic medication 1 h prior to the experiment. Compliance with this procedure was systematically verbally confirmed by the examiner before data collection. All participants were in mild-to-moderate stages of PD, and data acquisition was conducted in a supervised hospital environment, with the clinical care team available on site throughout the procedure. Following this, participants were instructed to stand quietly on a force plate with their feet positioned hip-width apart and arms resting alongside their body, maintaining a distance of 1 m from a 40-in.-screen TV.

Standing posture was chosen as an action-oriented bodily condition rather than as a neutral experimental constraint. From an embodied cognition perspective, empathic engagement involves not only perceptual and affective processes but also bodily dispositions related to readiness for action and interaction with others (Gallese 2005). Standing facilitates the activation of sensorimotor systems and allows postural dynamics to be expressed during empathic engagement. This choice is particularly relevant in PD, where upright posture is known to elicit compensatory postural control strategies such as increased rigidity and reduced movement flexibility (Schmit et al. 2006). Assessing empathy under standing conditions therefore enables the examination of potential interactions between postural regulation and embodied empathic processes, which would be difficult to observe in more passive postures such as sitting.

To ensure participant safety and minimize physical demands, standing periods were brief (60 s) and interleaved with seated rest intervals (3 min). All procedures were conducted in a supervised hospital environment, with clinical staff available on site throughout data collection. These procedures are consistent with validated approaches for the safe assessment of autonomic and postural regulation in PD (Schmit et al. 2006; Asahina et al., 2014; Gonçalves et al. 2022), and no adverse events occurred during the study. The videos for each condition (pain and baseline) were randomly displayed on the screen while data on postural control and physiological responses were collected. After watching the videos, participants assessed their emotional perception in both experimental conditions, using the SAM (Bradley and Lang 1994). This 9-point scale (1–9) measures emotional responses to a stimulus across three dimensions: valence (ranging from ‘unpleasant’ to ‘pleasant’), arousal (from ‘low’ to ‘high’), and dominance (from ‘lack of control’ to ‘full control’). Higher scores indicate a more positive valence, greater arousal, and an increased sense of control, while lower scores reflect a more negative valence, lower arousal, and a diminished sense of control. Immediately after completing the self-report measures in the pain condition, a researcher conducted a phenomenological interview with the participant.

### Data collection

#### First-person: the phenomenological interview

All interviews were conducted in Spanish by the same researcher, recorded using an audio device, and later transcribed verbatim. To maintain methodological consistency, the interviews followed a standardized protocol inspired by the microphenomenological interview guidelines (Petitmengin 2006), ensuring uniformity in data collection. At the start of each interview, participants were asked to describe the videos and then choose the one that represented the most intense overall experience.

The interviews explored the multidimensional aspects of the empathy-for-pain experience—including bodily, emotional, motivational, and thought dimensions. An essential aspect of the interviews, in line with the phenomenological method, was ensuring that both the researcher and the participant, in suspending the ‘natural attitude,’ refrained from evaluative interpretations of the experience (Hamilton et al., 2018; Petitmengin et al. 2019). To facilitate this, the interviewer guided participants to vividly recall and describe their experience in detail, with particular attention to how these aspects emerged and were lived experientially from a first-person perspective. Consistent with core microphenomenological principles, the interviews focused on a single, concrete scene drawn from the video stimulus; were conducted in an evocation mode—i.e. guiding participants to re-situate themselves in the original experiential context—to support participants’ re-entry into the lived experience in a fine-grained manner; and relied on open, content-empty questions—i.e. questions devoid of predefined categories or interpretative cues—with iterative refinement to unfold the micro-dynamics of experience. Following Petitmengin (2006), interviewers systematically redirected participants away from general explanations or evaluations toward the experiential ‘how’ of the situation, encouraging a suspension of habitual judgments (epochè). The interview structure was organized around four predefined experiential domains—bodily, emotional, motivational, and thought—to facilitate a comprehensive exploration of empathic experience. Example prompts included “How does the sensation of tension feel?”, “How does that impulse to help the person feel?”, and “What are the words that appear in your mind?”. At the same time, the protocol constituted an adaptation rather than a full canonical microphenomenological interview: interviews were conducted in a single, time-bounded session, and the interview structure was defined *a priori* around four experiential domains relevant to empathy—bodily sensations, emotional experience, motivational tendencies, and cognitive or meaning-related processes. Within these domains, however, questioning remained open and non-inductive, preserving the core epistemic commitments of the microphenomenological approach. Examples of phenomenological interviews are provided in Supplementary Material I, available at: https://osf.io/nfvhd/overview.

#### Third-person: motor and physiological data collection

Postural movement data were recorded using a Bertec FP4060-05-PT stabilometric platform (Bertec Corporation, Columbus, Ohio, USA). Integration of all stabilometry signals was accomplished using a BIOPAC MP150 data acquisition and analysis system with AcqKnowledge software (BIOPAC Systems, Inc.). The electrocardiogram (ECG) was captured using disposable snap ECG electrodes in a modified lead II configuration, with one electrode placed beneath the right clavicle and the other near the lower ribs on the left side. Simultaneously, electrodermal activity (EDA) was recorded using disposable snap electrodes attached to the palmar surface of the distal phalanges of the first and second fingers.

A MATLAB (MathWorks, Natick, MA, USA) script was used to present the stimuli on the 40-in. screen and send signals to the AcqKnowledge software for synchronizing the presentation of stimuli with the stabilometry and physiological data. The ECG and EDA were also recorded and synchronized with the stimulus presentation via BIOPAC.

### Data analysis

#### First-person: phenomenological analysis

Data analysis followed Giorgi’s descriptive phenomenological psychological method (Englander 2016; Giorgi et al. 2017; Englander and Morley 2021). Giorgi’s approach focuses on identifying meaning units, defined as statements that express the participant’s direct experience and aim to capture the essence of what was lived (Giorgi 1997). These units are then grouped into subthemes and main themes to subsequently identify experiential structures. This structure is obtained through the systematic relation and integration of the main themes, moving from particular experiential aspects toward an essential and holistic understanding of how participants make sense of their lived experience (Giorgi et al. 2017). In addition, and to enhance the integrity and consistency of our phenomenological exploration, we adopted an iterative triangulation approach that included systematic quantitative assessment of inter-rater agreement throughout the analysis (Martínez-Pernía et al. 2023; Troncoso et al. 2024; Andreu et al. 2025). What follows is a detailed account of how phenomenological procedures and quality control measures were integrated to increase the robustness of the analysis.

Throughout the entire phenomenological analysis, all three analysts (D.M-P., A.Z., and D.P.) were fully blinded to the results of the quantitative measures (i.e. self-report, postural, and physiological measures). This blinding procedure was implemented specifically to prevent methodological circularity during the integration of phenomenological findings with quantitative data. Moreover, all three analysts have formal training in phenomenological interviewing and descriptive phenomenological analysis, ensuring methodological rigor and consistency.

The process began with each of the three analysts conducting an in-depth reading of the interview transcripts using Atlas.ti 9 (2022) qualitative data analysis software. Each researcher drafted a concise synopsis of the participant’s account to capture the essence of the described lived situation. Following this, key passages were identified and marked as ‘meaning units,’ representing segments that directly conveyed aspects of the participants’ lived experience. These expressions were then transformed into psychologically meaningful codes.

The initial coding system—and the broader analytical process leading to the elaboration of the experiential structure—was developed from the first set of 10 interviews and progressively refined through iterative comparison across subsequent cases. This included developing detailed phenomenological codes such as ‘body tension’ and ‘body anguish,’ which were subsequently grouped into higher-order conceptual categories like ‘body feeling,’ based on thematic convergence across cases. As the analysis unfolded, core themes such as ‘bodily resonance’ and their corresponding subthemes (e.g. ‘affective quality’) began to emerge. These were subject to collective deliberation among the research team to achieve conceptual clarity and agreement. This collaborative process allowed for the development of a shared structure of themes, subthemes, and categories, contributing to the analytic rigor and trustworthiness of the results. From this thematic framework, the overall structure of the experience was elaborated. By integrating the emergent components, the analysis moved from isolated experiential fragments toward more fundamental insights into participants’ lived understanding. This structural synthesis was achieved by systematically examining variations in experiential elements to uncover their essential interrelations. While the core abstraction was grounded in the initial interviews and refined until saturation was achieved, the process of review and adjustment continued throughout the remaining interviews.

Subsequently, each researcher independently conducted a case-by-case analysis using the established framework of structures, themes, subthemes, and categories. Once the qualitative stage was complete, the team proceeded to extract structured data from all the interviews for quantitative comparison. Inter-rater agreement was continuously assessed throughout the triangulation process using percentage agreement at multiple analytical levels (categories, subthemes, themes, and experiential structures). This procedure allowed the systematic identification of convergences and discrepancies across coders. Analytical units displaying full agreement (100%) were retained without further discussion, whereas units with lower agreement were subjected to targeted triangulation and discussion until consensus was reached. In line with the consensus-oriented and iterative nature of the analysis, agreement indices were used to guide triangulation and discussion rather than to estimate independent pre-consensus reliability. This iterative process resulted in full convergence across all interviews and analytical levels in the final coding framework. To enhance accessibility and transparency, agreement patterns and triangulation outcomes were visualized throughout the analysis using Microsoft Fabric v.F1 and presented through an interactive dashboard integrated into a dedicated web platform (https://5elab.cl/spectrum-embodied-intersubjective-synchrony-empathy/).

This multistep procedure was applied systematically to each participant’s data and constituted a fully triangulated analytical process.

#### Third-person: motor and physiological data analysis

Posturographic, cardiac, and electrodermal signals were recorded continuously throughout the video projection for each condition. However, all analyses were performed on temporally segmented data at the scene level rather than on the full 60-s montage. For each scene, a central 6-s segment was extracted, excluding scene onset and inter-scene transition periods. These segments were selected to capture responses temporally proximal to the core event of each scene while minimizing contamination from montage boundaries. All physiological and postural metrics described below were computed within these 6-s segments and subsequently averaged across scenes within each condition.

##### Postural movement

Postural control was assessed using a stabilometric force platform, which allows continuous measurement of ground reaction forces and COP dynamics as established indicators of postural regulation. Posturographic data were acquired using a force platform, providing stabilometric force components (Fx, Fy, Fz) and moment components (Mx, My, Mz) at a sampling rate of 125 Hz. The COP was calculated in the antero-posterior (COPy) and medio-lateral (COPx) directions. COP time series from each 6-s segment were low-pass filtered using a fourth-order Butterworth filter with an 8-Hz cutoff. Power spectral density was estimated using Welch’s method. Extracted COP features included resultant mean velocity and total path length.

##### Physiological data

Heart rate was recorded at 500 Hz. R peaks were detected in MATLAB using the Pan–Tompkins algorithm (Sedghamiz 2023). HR values were computed within each 6-s segment and averaged across scenes within each condition.

Electrodermal activity was sampled at 500 Hz. The phasic component was extracted using a convex optimization approach (Greco et al., 2016). Mean phasic activity was computed within each 6-s segment and averaged across scenes within each condition.

#### Integrative statistical analysis

Two different statistical approaches were employed to examine ways in which phenomenological results revealed underlying patterns within the quantitative data. In both analyses, data were grouped according to the experiential structures identified in the phenomenological analysis, allowing for statistical comparisons and insight into their quantitative distinctions.

The first approach involved analysis of mood and social cognition scales, as well as the SAM dimensions (valence, arousal, and dominance). To select the most appropriate statistical tests, data distribution was assessed for normality (Shapiro–Wilk test) and homoscedasticity (Levene’s test). When both assumptions were met, independent samples *t*-tests were conducted. If normality was violated, regardless of homoscedasticity, the Mann–Whitney *U* test was applied.

The second approach examined the effects of Condition (pain versus baseline) and Structure of Experience. Prior to inferential analyses, all posturographic, cardiac, and electrodermal dependent variables were assessed for normality (Shapiro–Wilk test) and homogeneity of variance (Levene’s test). COP-derived measures, HR, and phasic EDA exhibited positively skewed distributions and/or variance heterogeneity. Accordingly, all these variables were log10-transformed prior to statistical analysis to better satisfy model assumptions.

A 2 × 2 mixed-design ANOVA was performed to assess the main effects of condition and structure of experience (identified from phenomenological data), as well as any interactions. Because the within-subject factor included only two levels, the assumption of sphericity was automatically satisfied and no Greenhouse–Geisser correction was applied (ε = 1). When significant main or interaction effects were detected, *post hoc* comparisons with Bonferroni correction were conducted to identify pairwise differences.

Outlier detection was performed separately for each dependent variable, condition, and group using a robust interquartile range–based criterion. Observations exceeding 3 × IQR from the first or third quartile were classified as extreme values and excluded prior to inferential analyses. The number of excluded observations per dependent variable, condition, and structure of experience is reported in Supplementary Table 1, available at: https://osf.io/nfvhd/overview.

Effect sizes for ANOVA results are reported as generalized eta squared (η^2^_G). All statistical analyses were conducted using R Studio (R Core Team 2020), with the significance level set at α = 0.05 for all tests.

## Results

*Demographic distributions*. The present study analyzed a sample of 42 individuals diagnosed with PD, with a gender distribution of 19 females (45.2%) and 23 males (54.8%). The mean age of participants was 70.36 ± 6.34 years, and the mean years of formal education was 13.36 ± 4.57 years. Regarding cognitive assessments, the mean MMSE score was 28.00 ± 1.81.

*Order of reporting*. In the sections to follow, we first present the phenomenological results, focusing on the experiential structures. We next report their integration with self-report questionnaires and, subsequently, their integration with motor and physiological data. Finally, we provide a detailed description and explanation of Parkinson’s Empathy Bodyssence, offering a more holistic and comprehensive interpretation by integrating both qualitative and quantitative data.

As noted previously, although the phenomenological data from this study have already been published, this result section presents the overarching experiential structures that emerged from the qualitative analysis. These structures constitute the qualitative dimension to be associated with quantitative measures, including self-report, motor, and physiological data. For a more detailed account of lower-order levels of abstraction, see the codebook in Supplementary Material I, available at: https://osf.io/nfvhd/overview.

### Experiential structures

**Figure 1:**
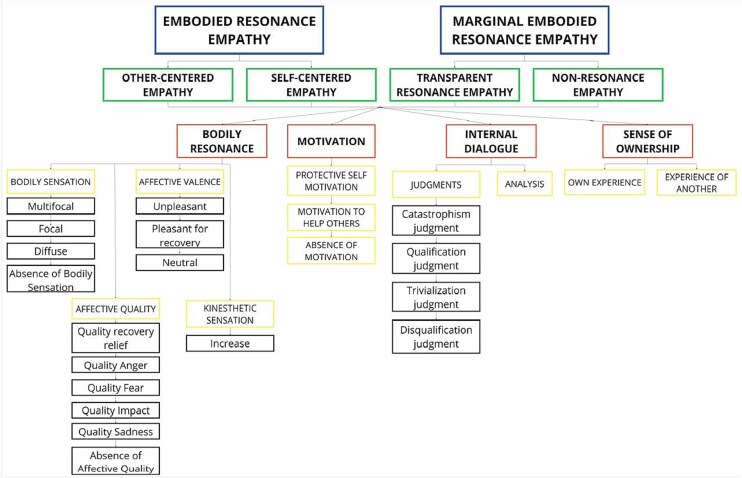
Graphical representation of the experiential categories. Categories highlighted in blue indicate the overarching experiential structures. Categories in green boxes indicate the intermediate experiential structures. Categories highlighted in red indicate the main themes, those in yellow denote subthemes, and categories in regular black represent the subcategories derived from participants’ narratives.

The phenomenological analysis gave rise to a structured, hierarchical organization of participants’ lived experiences, progressing through multiple levels of abstraction (Fig. 1). At the initial level of coding, 43 meaningful experiential codes were identified (e.g. chest pain), capturing fine-grained descriptive elements across narratives. These were synthesized into 18 experiential subcategories (e.g. focal sensations), which reflected recurring experiential patterns. The subcategories were subsequently organized into broader subthemes according to conceptual affinity (e.g. bodily sensations) and then grouped under four core themes that represented fundamental experiential dimensions with a transversal and relational character: bodily resonance, motivation, internal dialogue, and sense of ownership. These four main themes were further integrated into four intermediate experiential structures: Other-Centered Empathy, Self-Centered Empathy, Transparent Resonance Empathy, and Non-Resonance Empathy—each reflecting a distinct mode of experiencing empathy, as articulated through the participants’ first-person accounts. These modes express distinct ways in which empathic experience was embodied and experienced across participants. Finally, for each participant, empathy for pain manifested as one of two overarching experiential structures: Embodied Resonance Empathy (*n* = 25) and Marginal Embodied Resonance Empathy (*n* = 17). Below, we provide a detailed account of the characteristics and experiential dynamics that define each of these two structures. For a review of examples of the complete interviews by structure, see Supplementary Material I, available at: https://osf.io/nfvhd/overview.

#### Embodied Resonance Empathy

Embodied Resonance Empathy emerged as an experiential structure in which participants reported engaging with another’s suffering through bodily, emotional, and kinesthetic responses that are intrinsically connected to the perceived experience of the other. This connection manifests as a congruence between internal sensations—such as visceral and muscular responses—emotional states and kinesthetic impulses—all tightly coupled with the perceived suffering of the other. Participants also reported spontaneous evaluative or meaning-related thoughts about the situation being observed (e.g. concerning what was happening or how the situation was unfolding), co-present with bodily sensations and emotions as part of the lived experience. The experience is characterized by kinesthetic impulses that prompt action in response to the other’s pain. These impulses arise alongside strong bodily sensations and intense emotions, such as grief, fear, anxiety, or distress. Whether individuals feel a compelling urge to move toward the other, driven by the need to comfort or assist, or a powerful impulse to withdraw due to overwhelming discomfort, both responses are grounded in a personally distressing engagement and a deeply felt internal stance toward the suffering being witnessed. They took place within an experiential flow in which bodily sensations, emotions, and evaluative content are co-present.

“I shrank completely because I saw that he was going to fall and that I couldn’t stop the accident, so I felt nervous, like I was moving a lot, anticipating something fatal, something really bad, really bad […] I felt tightness all over my body, especially in my hands and arms […] then, at the moment of the fall, I felt something in my chest, I had to take a deep breath, everything felt tight […] I felt sadness, anguish… how can I save him, I wish I knew what to do, I thought… a desire to help him.” (P50)

“I knew that at some point he was going to fall […] and my body felt a horrible anguish, everything contracted—my legs, my arms […] I felt it as if it were happening to me, with the athlete […] the tension I feel is the anguish and the fear, the urge to be able to do something for him… I don’t know, the urge to stop that truck and tell him no, that they are a danger […] I felt my whole body stiff, with my hands clenched tight.” (P34)

“Anguish—I suffer the impact […] in my shoulders, in my head I feel a weight …, I suffer everything that comes with a fall […] here I watched the scene because I had to, but I tried not to watch or to shift, I tried to avoid the jumps.” (P4)

#### Marginal Embodied Resonance Empathy

Marginal Embodied Resonance Empathy refers to an experiential structure in which individuals engage with another’s suffering while experiencing reduced bodily resonance, characterized by a lack of bodily sensations and, in some cases, an emotionally neutral state. Instead of responding through bodily–affective synchrony, their engagement is primarily mediated by external visual cues, shaping their perception and understanding of the event. Participants in this structure do not experience bodily sensations in response to the suffering of the other. While some still register unpleasant emotions—such as grief, fear, anxiety, or worry—their experience remains grounded in external perception rather than an internally felt bodily response. Others, however, describe their emotional state as neutral, without distress or emotional involvement. This reduction in bodily resonance is also accompanied by a diminished tendency to act, as individuals do not experience a kinesthetic impulse to move in response to the suffering of the other. Their stance tends to remain oriented from their own experiential position, observing the event from a self-contained perspective rather than through a shared bodily resonance. Participants also report evaluative or meaning-related thoughts focused on the situation being observed (e.g. assessing the activity, its risks, or its execution), unfolding in the absence of salient bodily or affective resonance. Their engagement remains passive and observational, often focused on analyzing the technical aspects of the activity, rather than on internal bodily or affective reactions.

“I was fine, just watching calmly, and then he slipped… I said to myself, I wouldn’t do that, I don’t want to end up broken. That’s what I thought. I felt sorrow when I saw him fall, […] but without any bodily sensation. I just thought of him—the person who falls—and imagined he must not have ended up well at all. I also thought about the foolish thing he did.” (P7)

“Participant: I realized he was going to fall. I noticed it because I started paying attention to the skis. His skis came together, they crossed, and when that happens it is very dangerous—at that moment, I thought he was going to fall. Interviewer: How was your experience when the person falls? Participant: when he falls I don’t really feel anything, no, no, I don’t feel anything. Of course, it’s unfortunate what is happening to the guy, but feeling something in my body, no… I thought about the fact that I have always skied, but I never took more risks than appropriate; I never fell badly. Interviewer: Did you feel any urge to do something during the video? Participant: No, nothing.” (P6)

“I kept thinking about how stupid the guy was—it was obvious the risk of losing control was too high. I kept asking myself, can someone really be that stupid? I knew he was going to fall, it was clear—he was getting tired, and you could see it. When he actually fell, I felt a kind of anxiety, mixed with disbelief.” Interviewer: “What do you mean by anxiety?” Participant: “It was more like a thought—a reflexive reaction—but it didn’t manifest in my body. And when he finally fell… I just confirm that he was wrong, that what he did was a mistake… I didn’t feel any urge to do something.” (P31)

“The one where he climbs between two walls… I kept thinking about the forces, the speed—everything that made it obvious there was no way it would end well […]. And when I saw him fall, my body didn’t react at all. It felt like I disconnected, like I stopped paying attention, thinking this is just foolish […] it didn’t cause me fear or tension in my body, only the idea that he had chosen badly, that it was too risky […] if he had put a mattress underneath it would have been different, but where he fell everything was dirty, with logs, a place to break himself completely.” (P21)

### Integration of experiential structures with self-report measures

To analyze the relationship between the two identified experiential structures and participants’ affective and cognitive responses, we conducted a comparative analysis. Table 1 presents descriptive statistics for all variables across the two experiential groups.

**Table 1:** Descriptive statistics for mood, social cognition, and emotional perception variables by experiential structure.

Variable	Marginal embodied resonance empathy	Embodied resonance empathy
GDS	3.88 ± 2.91	5.64 ± 3.03
GAD-7	5 ± 3.72	7.32 ± 4.75
Mini-SEA	21.96 ± 3.21	22.28 ± 3.29
IRI	52.24 ± 27.5	52.8 ± 26.52
SAM_Valence	4.18 ± 1.88	2.84 ± 1.99^*^
SAM_Arousal	6.24 ± 1.75	7.4 ± 1.44^*^
SAM_Control	6.88 ± 1.76	6.4 ± 2.18

The table presents the mean values and standard deviations of different measures of emotional and cognitive processing related to empathy, social cognition, and affective states, grouped according to the phenomenological structure. GDS, Geriatric Depression Scale; GAD-7, Generalized Anxiety Disorder; Mini-SEA, Mini-Social Cognition & Emotional Assessment; IRI, Interpersonal Reactivity Index; SAM, self-assessment manikin. ^*^P-value < .05.

**Figure 2:**
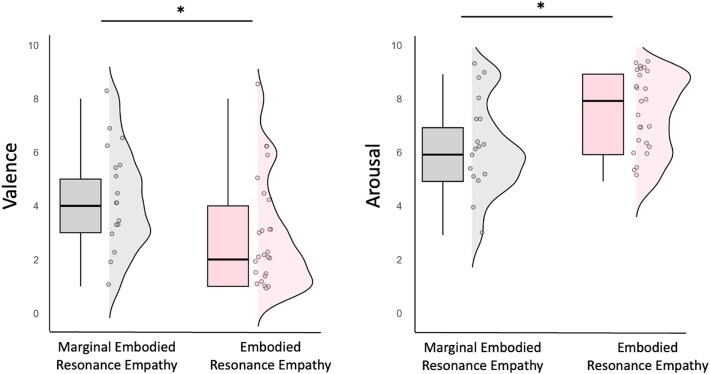
Differences in SAM valence and arousal between experiential structures. This figure presents boxplots with density maps, alongside individual data points, illustrating differences in valence (left) and arousal (right) between the Marginal Embodied Resonance Empathy (*n* = 17) and Embodied Resonance Empathy (*n* = 25) groups. The density maps display the distribution of the data, the boxplots represent the median, interquartile range, and data variability, and the individual points indicate each participant’s score. Participants with Embodied Resonance Empathy (pink) reported lower valence scores—indicating a more negative emotional experience—and higher arousal scores, reflecting greater emotional activation, compared to those with Marginal Embodied Resonance Empathy (gray). ^*^P < .05.

Although all mood, social cognition, and emotional perception variables were included, only some yielded statistically significant differences between groups. Statistically significant differences emerged specifically in the SAM dimensions of valence and arousal. For SAM_Valence, the analysis showed a statistically significant difference between the two groups, Embodied Resonance Empathy and Marginal Embodied Resonance Empathy (*U* = 123.00, *Z* = −2.326, *P* = .021, *r* = −0.36), indicating that participants who experienced Embodied Resonance Empathy reported significantly more unpleasant affect than those in the Marginal Embodied Resonance Empathy group (Fig. 2). For SAM_Arousal, the Mann–Whitney *U* test also revealed a significant difference (*U* = 294.00, *Z* = −2.133, *P* = .034, *r* = −0.33), suggesting that those whose response was characterized by Embodied Resonance Empathy experienced higher arousal than those whose experience was characterized by Marginal Embodied Resonance Empathy (Fig. 2). These findings suggest that individuals characterized by Embodied Resonance Empathy exhibited greater affective engagement, as evidenced by more unpleasant emotional responses (lower valence) and heightened arousal during the empathy-for-pain condition. This pattern aligns with deeper emotional resonance and embodied attunement to the suffering of others. In contrast, those in the Marginal Embodied Resonance Empathy group reported more neutral or pleasant affect (higher valence) and showed lower emotional arousal, indicating reduced embodied resonance and a more observational mode of engagement. This mode was characterized by attending to technical aspects of the sporting action or making evaluative judgments, rather than by a felt connection with the other’s suffering.

### Integration of experiential structures with motor and physiological activity

In this subsection, we compare motor and physiological activity across conditions (baseline and pain) and experiential structures. For a statistical comparison of the entire sample by condition, refer to the Supplementary Material I, available at: https://osf.io/nfvhd/overview.

#### Postural movement

**Figure 3:**
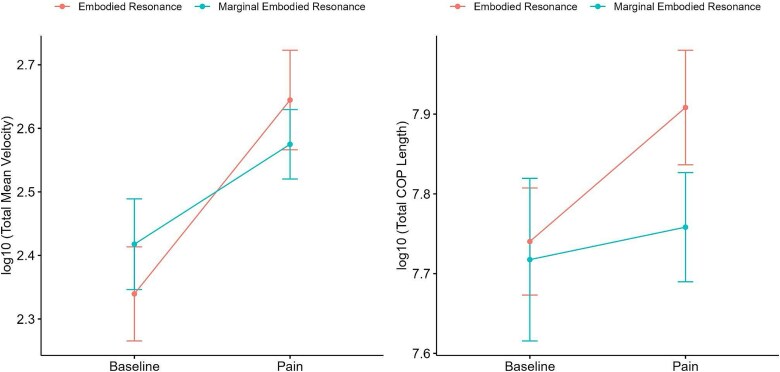
The figure presents the results of the total mean velocity (log-transformed) and total length (log-transformed) comparison between the *Pain* and *Baseline* conditions, grouped by experiential structure. The bars represent the means of total mean velocity, with error bars indicating the standard deviation.

A 2 × 2 mixed-design ANOVA was conducted with Structure of Experience (embodied versus marginal resonance empathy) as a between-subject factor and Condition (pain versus baseline) as a within-subject factor. The analysis revealed a significant main effect of Condition, *F*(1,40) = 47.76, *P* < .001, with a moderate effect size (η^2^_g = 0.106), indicating that postural movement velocity differed significantly between the Pain and Baseline conditions. However, there was no significant main effect of Structure of Experience, *F*(1,40) = 0.00, *P* = .967, suggesting that this factor alone did not influence postural movement velocity. A significant Condition × Structure of Experience interaction was observed in Total Mean Velocity of Postural Movement (log-transformed), *F*(1,40) = 4.88, *P* = .033, with a small effect size (η^2^_g = 0.012). This finding indicates that the effect of Condition on postural movement velocity varied depending on the Structure of Experience (Fig. 3). *Post hoc* pairwise comparisons further revealed that postural movement velocity significantly differed between Pain and Baseline conditions within the marginal embodied resonance empathy (*P* = .01) and even more strongly within the embodied resonance empathy (*P* < .001). However, there were no significant differences between both experiential structures within either the Pain condition (*P* = .512) or the Baseline condition (*P* = .471). Taken together, Pain conditions elicited significantly greater postural movement velocity than Baseline conditions. Importantly, the Condition × Structure of Experience interaction suggests that this increase in postural movement velocity was stronger in the Embodied Resonance Empathy structure than in the Marginal Embodied Resonance Empathy structure.

A 2 × 2 mixed-design ANOVA on Total Length was conducted with Structure of Experience and Condition as factors. The analysis revealed a marginal main effect of Condition, *F*(1,39) = 4.11, *P* = .050, with a small effect size (η^2^_g = 0.022), suggesting that Condition may influence Total Displacement. However, there was no significant main effect of Structure of Experience, *F*(1,39) = 0.77, *P* = .386. Additionally, the Condition × Structure of Experience interaction was not significant, *F*(1,39) = 1.53, *P* = .224 (η^2^_g = 0.008). Although the interaction effect did not reach statistical significance, exploratory *post hoc* pairwise comparisons revealed differential patterns depending on the Structure of Experience. Specifically, Total Length significantly increased in the Pain condition compared to the Baseline condition within the Embodied Resonance Empathy group (*P* = .003), whereas no significant difference was observed within the Marginal Embodied Resonance Empathy group (*P* = .694). These results indicate that while Condition had a significant effect on Total Length, the increase was more pronounced within the Embodied Resonance Empathy structure, suggesting greater motor reactivity in response to the Pain condition. These interpretations should be made with caution given the lack of a significant interaction effect in the overall model and the marginal significance of the Condition main effect.

#### Physiological activity

A 2 × 2 mixed-design ANOVA was conducted on Heart Rate (log-transformed) with Structure of Experience and Condition as factors. The analysis revealed no significant main effects for Structure of Experience, *F*(1,36) = 1.07, *P* = .308, or Condition, *F*(1,36) = 0.68, *P* = .413. Additionally, the Condition × Structure of Experience interaction was not significant, *F*(1,36) = 0.39, *P* = .538, indicating that the effect of Condition on Heart Rate did not vary depending on the Structure of Experience.

A 2 × 2 mixed-design ANOVA was also conducted on EDA (log-transformed) with the same factors. The analysis revealed no significant main effects for Structure of Experience, *F*(1,20) = 2.27, *P* = .147, or Condition, *F*(1,20) = 0.87, *P* = .362. The Condition × Structure of Experience interaction was also not significant, *F*(1,20) = 0.03, *P* = .876, indicating that the effect of Condition on EDA did not vary depending on the Structure of Experience.

### Parkinson’s Empathy Bodyssence

Finally, in this subsection, we present a detailed description and analysis of Parkinson’s Empathy Bodyssence in the context of empathy for pain, integrating both qualitative and quantitative data to provide a holistic and comprehensive interpretation. Following the definition proposed by Troncoso et al. (2024), Empathy Bodyssence is an embodied process that constitutes an integrative structure of both neurophysiological and subjective attributes of consciousness. This concept encapsulates how participants physically and emotionally engage with the suffering of others, highlighting the dynamic interplay between sensorimotor activity, affective modulation, motivational dynamics, and cognitive processes.

Based on the integration of phenomenological and quantitative (motor and physiological) data (see Supplementary Material II for access to the full dataset, available at: https://osf.io/nfvhd/overview), two distinct structures of Parkinson’s Empathy Bodyssence emerged. These structures reflect differing ways in which individuals with PD embodied empathy for pain: one marked by visceral, affective, and action-oriented engagement, and the other characterized by a more detached, visually guided, and cognitively interpreted form of empathic response.

#### Resonance Bodyssence

**Figure 4:**
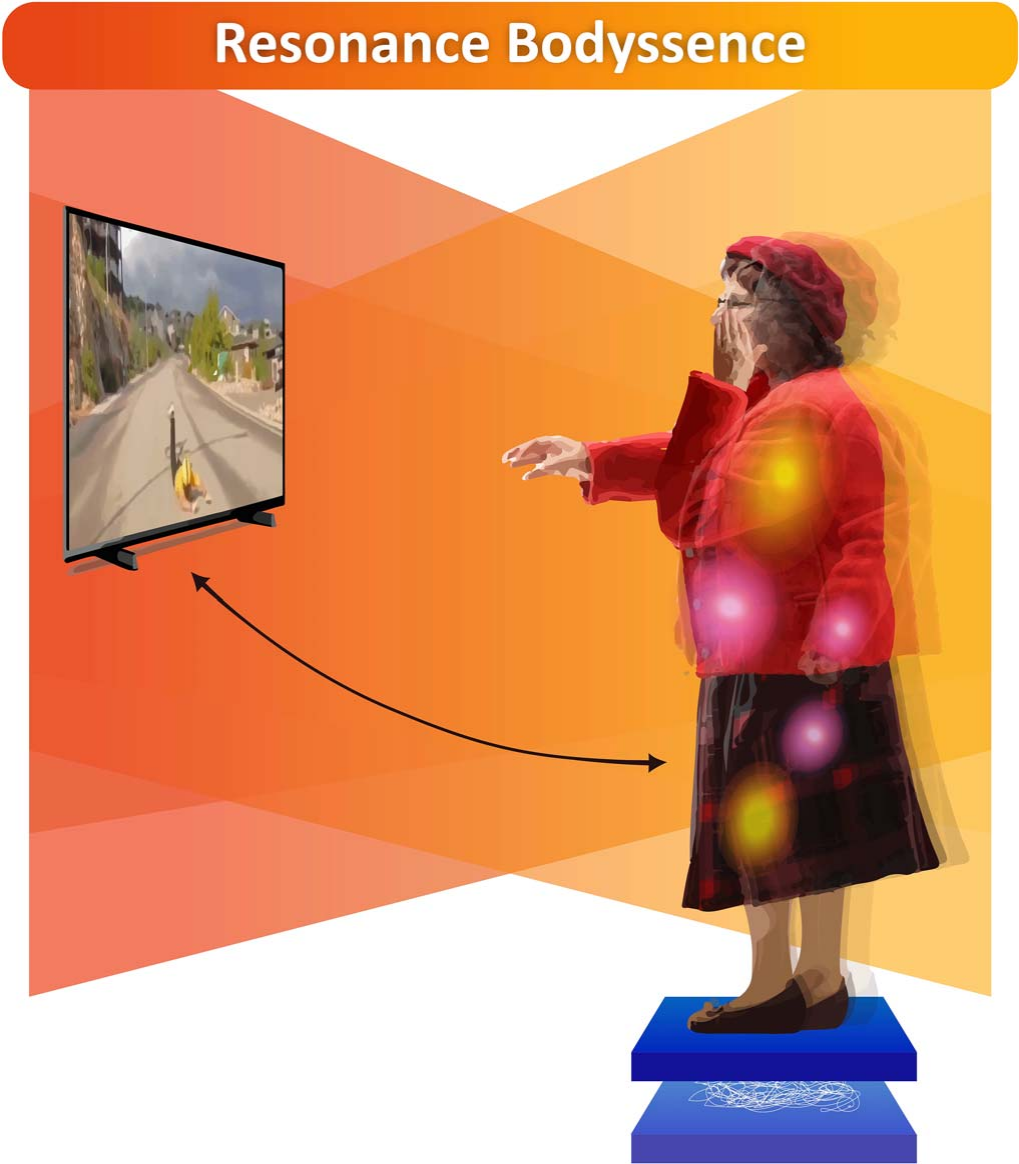
Illustration of the Resonance Empathy Bodyssence structure, characterized by strong bodily–affective engagement during empathic experience. The vibrant orange-red color gradient extending toward the athlete’s suffering represents a strong embodied attunement between the Parkinson’s patient and the pain of the other. The yellow glowing spots indicate areas of visceral and muscular activation, while the pink lights reflect unpleasant emotional responses triggered by witnessing the other’s pain. The participant’s posture and reaching gesture express a kinesthetic impulse to act. The visible body outline and tremor suggest increased postural movement, and the stabilometric trace below shows enhanced displacement, reflecting amplified motor reactivity in the empathy-for-pain condition.

Resonance Empathy Bodyssence was characterized by a high degree of bodily–affective engagement, in which visceral sensations, muscular tension, and kinesthetic impulses emerged in direct connection with the other’s suffering. These bodily responses were intimately intertwined with emotional states such as grief, fear, or anxiety, forming a unified experiential pattern in which emotional intensity and sensorimotor responsiveness were integrated into a coherent empathic process. This embodied-affective resonance was reflected in increased postural reactivity, as evidenced by significantly higher mean velocity and total displacement in the empathy-for-pain condition. The urge to act—whether to approach or withdraw—was aligned with these bodily dynamics, reinforcing a sense of attunement to the suffering of the other (Fig. 4).

#### Marginal Resonance Bodyssence

**Figure 5:**
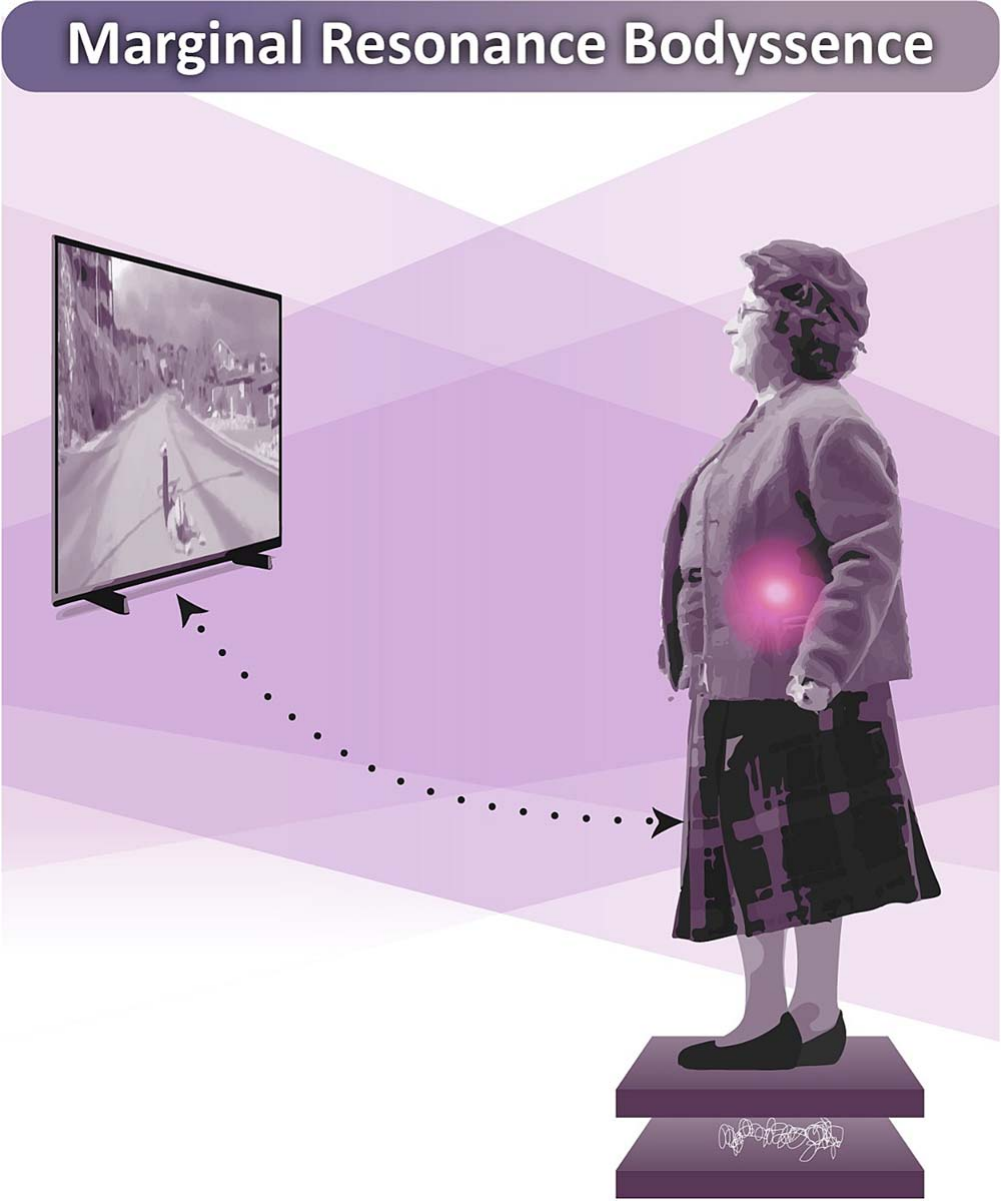
This figure illustrates the Marginal Resonance Bodyssence structure, characterized by a reduced level of bodily–affective engagement during empathic experience. The soft violet gradient projecting toward the athlete’s suffering represents a weakened connection between the Parkinson’s patient and the pain of the other. The pink light indicates the presence of unpleasant emotions in the absence of bodily resonance. The upright, static posture reflects the lack of kinesthetic impulses or motor readiness to respond. This diminished embodied engagement is further evidenced by the mirrored stabilometric trace below, which shows a smaller change in postural displacement, indicating reduced motor reactivity during the empathy-for-pain condition.

This structure was characterized by the absence of bodily sensations in response to the suffering of others. While some individuals still reported unpleasant emotions—such as grief, fear, anxiety, or worry—described during the phenomenological interview, these emotions were not accompanied by felt bodily responses. Instead, the empathic experience was guided by external visual cues, shaping how the situation was perceived and interpreted. Others described their emotional state as neutral, with no significant distress or affective engagement. This lack of bodily resonance was reflected in lower postural engagement in the empathy-for-pain condition, as indicated by a smaller increase in mean velocity and no significant change in total displacement. The absence of kinesthetic impulses to act further reinforced a passive and observational stance, where individuals did not experience a bodily drive to respond (Fig. 5).

## Discussion

This study aimed to integrate phenomenological analysis with self-report, motor, and physiological data through a neurophenomenological approach, in order to test hypotheses about distinct forms of embodied empathy for pain in PD. Classifying self-report, motor, and physiological data according to phenomenological structures revealed two distinct patterns of empathic engagement. Within this framework, the profile labeled Resonance Bodyssence, characterized by strong affective and somatic resonance, was accompanied by increased postural reactivity. This mode of engagement involved not only bodily–affective resonance but also the co-presence of meaning-related and evaluative aspects of the situation, experientially integrated with sensorimotor and affective processes. This response aligns with prior research demonstrating consistent activation of somatosensory and motor systems during the observation of others’ pain (Avenanti et al. 2005; Keysers et al. 2010; Betti and Aglioti 2016), highlighting that social interaction is experienced through the body and movement, thereby constituting a basic mechanism of social cognition (Colombetti 2014; Gallese 2014).

In contrast, the Marginal Resonance Bodyssence profile, defined by less embodied experiences, was associated with attenuated postural modulation. Here, empathic engagement was characterized by a more externally oriented and observational stance, in which evaluative and meaning-related aspects of the situation were present but unfolded with limited bodily–affective coupling. This pattern suggests disruptions in action–perception coupling mechanisms, which are essential for synchronizing the perception of another’s suffering with bodily response (Eddy and Cook 2018; Riečanský and Lamm 2019). These disruptions align with studies documenting sensorimotor deficits in emotional perception in PD (Bellot et al. 2021), possibly linked to altered patterns of connectivity between brain regions critical to social cognition, such as the premotor cortex, insula, and basal ganglia (Disbrow et al., 2014; Canu et al. 2015; Hu et al. 2015). Altogether, these findings suggest that empathic heterogeneity in PD does not arise merely from individual differences in subjective experience, but emerges as an integrated phenomenon in which sensorimotor, affective, and evaluative dimensions are deeply intertwined, jointly shaping and modulating empathic engagement.

On the other hand, the absence of differences in autonomic activity—both the total sample and between phenomenological structures—adds another layer of nuance. This finding aligns with literature describing autonomic dysfunction in PD (Asahina et al., 2014; Chen et al. 2020; Ozawa et al. 2024). Since empathy typically involves autonomic adjustments—variations in heart rate and skin conductance that reflect the simulation of observed affective states (Jankowiak-Siuda et al. 2011; Jauniaux et al. 2020; Sassenrath et al. 2021)—the attenuation of these responses in PD supports the hypothesis that autonomic regulation serves as a critical modulator of empathic engagement (Chauhan et al. 2008). At the same time, these null findings should be interpreted with caution. Given the modest sample size, the known variability and low signal-to-noise ratio of autonomic measures in PD, and the temporal and analytical constraints of the present design, it is possible that existing effects were smaller than the present study was powered to detect, or that alternative analytic strategies may be required to reveal them. Accordingly, the absence of autonomic differences should not be taken as evidence for the absence of autonomic involvement in embodied empathy, but rather as a methodological limitation that warrants further investigation. In this context, the observed dissociation between motor resonance and autonomic activation suggests that some patients may synchronize their posture with another’s suffering without proportional physiological activation, likely reducing the subjective intensity and depth of the empathic experience (Pick et al. 2019). It is worth noting, however, that factors such as clinical heterogeneity, dopaminergic treatment, and the sensitivity of measurement tools (e.g. temporal windows or laboratory contexts) may contribute to the absence of detectable effects; future studies should isolate these components to better determine the specific role of autonomic regulation in PD.

In recent years, the study of subjective experience has gained increasing relevance in cognitive science, progressively integrating into experimental designs in psychology and neuroscience (Berkovich-Ohana et al. 2020; Bitbol and Petitmengin 2013). In our study, we found a noteworthy complementarity between the stimulus-salience-based measure of emotional perception (SAM) and phenomenological structures. Given the statistically significant differences observed in valence and arousal, phenomenological analysis not only confirmed these findings but also offered a deeper, more situated interpretation. Rather than general patterns, phenomenological interviews revealed subtle differences in how participants made sense of their embodied emotional responses, adding granularity and context to the numerical scores (Varela 1996; Petitmengin 2006; Meadows 2024). This alignment illustrates how phenomenological methods can enrich self-report data by uncovering qualitative dimensions of lived emotional experience that are often obscured by purely quantitative approaches (Olivares et al. 2015;Gallagher and Zahavi 2020; Englander and Morley 2021). As such, integrating both methods strengthens ecological validity and provides a fuller picture of empathic processes in PD.

In this context, the observed convergence and divergence between phenomenological structures and self-report measures highlight the complementary yet non-overlapping nature of these approaches. While stimulus-salience-based measures of emotional perception, such as the SAM, showed significant differences aligned with phenomenological structures, trait-level empathy scales did not differentiate between groups. This pattern is consistent with previous work indicating that phenomenological methods are not primarily designed to capture stable dispositional traits, but rather to elucidate variations in lived, situated experience (Van Dam et al. 2009; Poletti et al. 2021; Troncoso et al. 2025). Trait questionnaires rely largely on higher-order, reflective processes, including generalized self-assessment and introspective judgments about empathic tendencies, detached from a specific experiential context (Petitmengin 2006). In contrast, phenomenological interviews are anchored in prereflective, moment-to-moment experience and focus on how empathy unfolds in relation to a concrete intentional object (Gallagher and Zahavi 2020; Englander and Morley 2021). From this perspective, the absence of differences in trait empathy or mood measures does not undermine the relevance of the phenomenological distinctions identified here; rather, it reinforces the idea that phenomenology captures qualitative, context-sensitive aspects of empathic engagement that standardized questionnaires may fail to detect. This complementarity strengthens the argument that phenomenological analysis provides access to dimensions of empathy that remain largely invisible to trait-based self-report instruments.

Taken together, these considerations motivate a broader methodological framing of the present study. More broadly, this methodological choice can be understood as an application of the Empirical 5E approach (Troncoso et al. 2023) and as part of a refinement of the neurophenomenological program itself. By foregrounding physiological, motor, and lived bodily dynamics alongside first-person reports, our approach responds to longstanding calls within embodied and enactive frameworks to move beyond strictly brain-centered explanations when addressing questions of embodiment (Varela et al. 1991; Thompson 2007; Colombetti 2014). In this sense, the present study illustrates how neurophenomenology can remain faithful to its original integrative commitment while expanding its empirical scope to include bodily and interactive processes that are central to the constitution of experience but are often underrepresented in neurophysiological designs.

An important aspect of these findings concerns the role of evaluative and moral appraisals during empathy for pain. Given that the stimuli depicted situations involving intentional risk-taking, participants across both experiential structures occasionally expressed judgments related to agency, responsibility, or competence. These evaluative processes were systematically coded within the Internal Dialogue theme (see Supplementary Material I) and were not specific to a single experiential structure. Rather, Resonance and Marginal Resonance Bodyssence differed in the experiential configurations within which such appraisals were embedded. Consistent with enactive accounts of cognition, evaluative appraisals in this context did not appear as separable cognitive additions, but as part of a situated sense-making process enacted through bodily engagement with the observed situation (Varela et al. 1991; De Jaegher and Di Paolo 2007; Colombetti 2014). In Resonance Bodyssence, evaluative judgments co-occurred with bodily–affective resonance, affective engagement, and motivational impulses, whereas in Marginal Resonance Bodyssence they tended to become experientially dominant and were accompanied by attenuated bodily sensation, neutral affect, and an externally oriented, observational stance. This distinction is further supported by the convergence between phenomenological structures and motor findings. Participants classified within Resonance Bodyssence exhibited increased postural reactivity, consistent with an embodied mode of empathic engagement characterized by enhanced bodily involvement during the observation of another’s pain (Troncoso et al. 2024). By contrast, the attenuated postural modulation observed in Marginal Resonance Bodyssence aligns with a more observational mode of engagement marked by reduced bodily involvement and limited action readiness (Schilbach et al. 2013; Fuchs 2017). Together, these results indicate that agency attribution and moral appraisal shape empathic engagement as integral components of embodied experiential configurations, whose impact depends on their integration with bodily–affective and motor dimensions during the empathic encounter.

A relevant aspect of the study’s findings lies in their clinical and therapeutic implications. The identification of differentiated embodied structures as a function of sensorimotor reactivity to others’ suffering suggests that interventions aimed at enhancing action–perception coupling could strengthen empathic resonance and, in turn, improve social interaction dynamics. In particular, movement-based therapeutic strategies, such as Argentine Tango, may be especially beneficial for patients presenting profiles of reduced embodied resonance, such as the Marginal Resonance Bodyssence. By emphasizing fluid movements, synchronous partner work, and postural balance control, tango addresses the motor–affective responses observed in this study, supporting both functional mobility and emotional well-being (Hackney and Earhart 2009; Hasan et al. 2022; Moț and Almăjan-Guță 2022; Whitehead et al. 2024). In this regard, for patients whose empathic engagement is predominantly expressed at an observational level, the interpersonal synchrony inherent to dance, which requires the dynamic coordination of movements, bodily sensations, and affective states with another, may function as a sensorimotor scaffold capable of reactivating processes of bodily–affective resonance. By fostering sustained enactive coupling with others, such practices may facilitate a shift from distant empathic involvement toward more embodied and participatory forms of engagement (Dieterich-Hartwell 2024; Heiberger et al. 2011). Additionally, although physiological reactivity was attenuated, consistent with autonomic blunting in PD, these signals constitute fundamental interoceptive indicators involved in accessing and modulating one’s own and others’ affective states (Jankowiak-Siuda et al. 2011). The observed attenuation of autonomic responses may therefore reflect a weakening of interoceptive processes that hinders deep, bodily grounded empathic engagement. In this context, programs focused on training interoceptive awareness and embodied cognition (Farb et al. 2007; Fogel 2009) offer a promising therapeutic avenue to help patients reconnect with and amplify these weakened internal signals, thereby supporting the restoration of empathic resonance. Together, these proposals underscore the need for an embodied perspective in neurorehabilitation, moving beyond an exclusive cognitive focus and including the restoration of bodily engagement as a core component of social interaction and affective attunement (Martínez-Pernía 2020; Håkstad et al. 2025).

### Limitations and future directions

This study presents several limitations. First, the quantitative sample size—while consistent with previous studies detecting autonomic and postural and autonomic modulation in PD (De Marinis et al. 2000; Kamieniarz et al. 2021)—could be increased to improve statistical power and enhance robustness. Second, the phenomenological analysis surpassed the point of theoretical saturation, meaning that no new experiential structures or significant dimensions emerged in the later stages of the analysis process (Petitmengin et al. 2019). However, increasing the number of interviews in future studies may help refine existing structures and capture greater experiential variability across different clinical profiles.

An additional limitation of the present study concerns the difficulty of dissociating, based on COP measures, postural modulations specifically related to empathic processing from those associated with the basal postural control required by standing posture. Although the experimental design incorporated strategies to attenuate this influence, such as analyzing intrasubject differences between conditions, assessing participants in the ON medication state, and strictly standardizing recording periods, standing posture necessarily entails continuous activation of balance control mechanisms. Consequently, some of the variations observed in COP may reflect automatic or compensatory motor adjustments not directly related to the empathic experience. Future studies could address this limitation by incorporating complementary muscular measures, such as electromyography, or by comparing different postural conditions, in order to more precisely discriminate between motor components and postural modulations associated with empathic processing. Future studies could examine whether empathy-for-pain stimuli depicting non–risk-related or non-intentional accidents give rise to different experiential and embodied patterns of empathic engagement. Systematically varying stimulus characteristics such as intentionality and perceived agency may help clarify how distinct forms of embodied resonance are enacted across contexts in PD.

Another limitation concerns data loss in EDA, potentially related to the typical motor fluctuations observed in PD, which are known to compromise the stability of physiological recordings in these clinical populations (Barrachina-Fernández et al. 2023). Increasing the sample size in future studies may help mitigate this issue by compensating for artifacts caused by involuntary motor activity, thereby enabling a more accurate exploration of the relationship between autonomic responses and the phenomenological structures of empathic experience. In addition, a more detailed clinical characterization of the sample was not systematically available for all participants. Specifically, information regarding motor severity (e.g. UPDRS-III), PD phenotypes, and levodopa equivalent daily dose could not be consistently reported. Future studies would benefit from incorporating a more comprehensive and standardized clinical profiling to better account for disease heterogeneity and its potential influence on postural and autonomic measures. Finally, the absence of a healthy control group limits the extent to which the observed experiential structures can be interpreted as specific to PD. While the present study was designed to characterize heterogeneity within a PD sample, future studies including age-matched control groups will be necessary to determine whether these embodied patterns of empathic engagement are specific to PD or reflect more general forms of variability in embodied empathy. Taken together, these considerations position the present work not only as a characterization of current limitations, but as a programmatic foundation for future neurophenomenological research.

## Conclusion

This study shows that empathy in PD is not a uniform capacity but is enacted through distinct embodied configurations that organize bodily, affective, and sense-making engagement. Using a neurophenomenological approach, we characterized how empathic experience unfolds as a lived, dynamic process emerging from the coupling between bodily sensations, affective resonance, and meaning-making.

Within this framework, Empathy Bodyssence refers to an enacted mode of organization rather than a discrete component of empathy, capturing how sensations, emotions, and action tendencies are jointly structured in experience. By integrating phenomenological structures with motor, physiological, and self-report measures, we identified differentiated patterns of empathic engagement: Resonance Bodyssence, marked by strong embodied and postural involvement, and Marginal Resonance Bodyssence, characterized by a more observational and bodily attenuated mode of engagement.

These findings highlight the value of phenomenology as an organizing level of analysis for making sense of interindividual variability in embodied and physiological responses, and they contribute to a more nuanced, enactive understanding of empathy in PD as a heterogeneous and dynamically enacted phenomenon.

## Supplementary Material

niag010_Supplementary_materials_SM_I

niag010_Supplementary_materials_SM_II

niag010_Supplementary_materials

## Data Availability

To ensure transparency and facilitate the review of our neurophenomenological framework, supplementary materials are available on the Open Science Framework (OSF) at https://osf.io/nfvhd/overview. This repository includes the quantitative dataset, the phenomenological codebook, and a selection of two examples of phenomenological interviews translated into English. In addition, detailed individual phenomenological analyses and systematic inter-rater agreement assessment can be accessed at https://5elab.cl/spectrum-embodied-intersubjective-synchrony-empathy/.
