# Supplementary material for: When the body resonates with the pain of the other: Empathy Bodyssence in Parkinson’s disease
Source: Neurosci Conscious. 2026 Apr 7;2026(1):niag010. doi: 10.1093/nc/niag010 (PMC13064857; doi:10.1093/nc/niag010)
Supplement: niag010_Supplementary_materials_SM_I [file niag010_supplementary_materials_sm_i.docx]

**Supplementary material**

## Third-person results

In this subsection show the effect of Condition (pain vs baseline) in the total sample. A repeated measures ANOVA was conducted to examine the effect of the condition on the dependent variables, without considering the structure of experience. The data were log-transformed to ensure normality of residuals. Post-hoc comparisons using the Bonferroni correction were performed.

The statistical results show differences in COP total mean velocity (log-transformed), showing a significant effect of condition, F(1,45)=51.15, p<.001, with the pain condition resulting in greater velocity compared to the baseline condition. A pairwise t-test further confirmed this difference, revealing a statistically significant increase in COP velocity in the pain condition relative to the baseline condition (p<.001) (Figure S1). Complementarily, the one-way ANOVA shows a significant effect of condition on COP displacement (log-transformed), F(1,47)=7.67, p=.009, with the pain condition resulting in greater displacement compared to the baseline condition. A pairwise t-test further confirmed this difference, revealing a statistically significant increase in COP displacement in the pain condition relative to the baseline condition (p=.009) (Figure S2).

A repeated measures ANOVA was conducted to assess the differences in heart rate (log-transformed), revealing no significant effect of condition on heart rate, F(1,42)=1.34,p=.253. Similarly, there was no significant effect of condition on electrodermal activity, F(1,38)=1.27, p=.239.


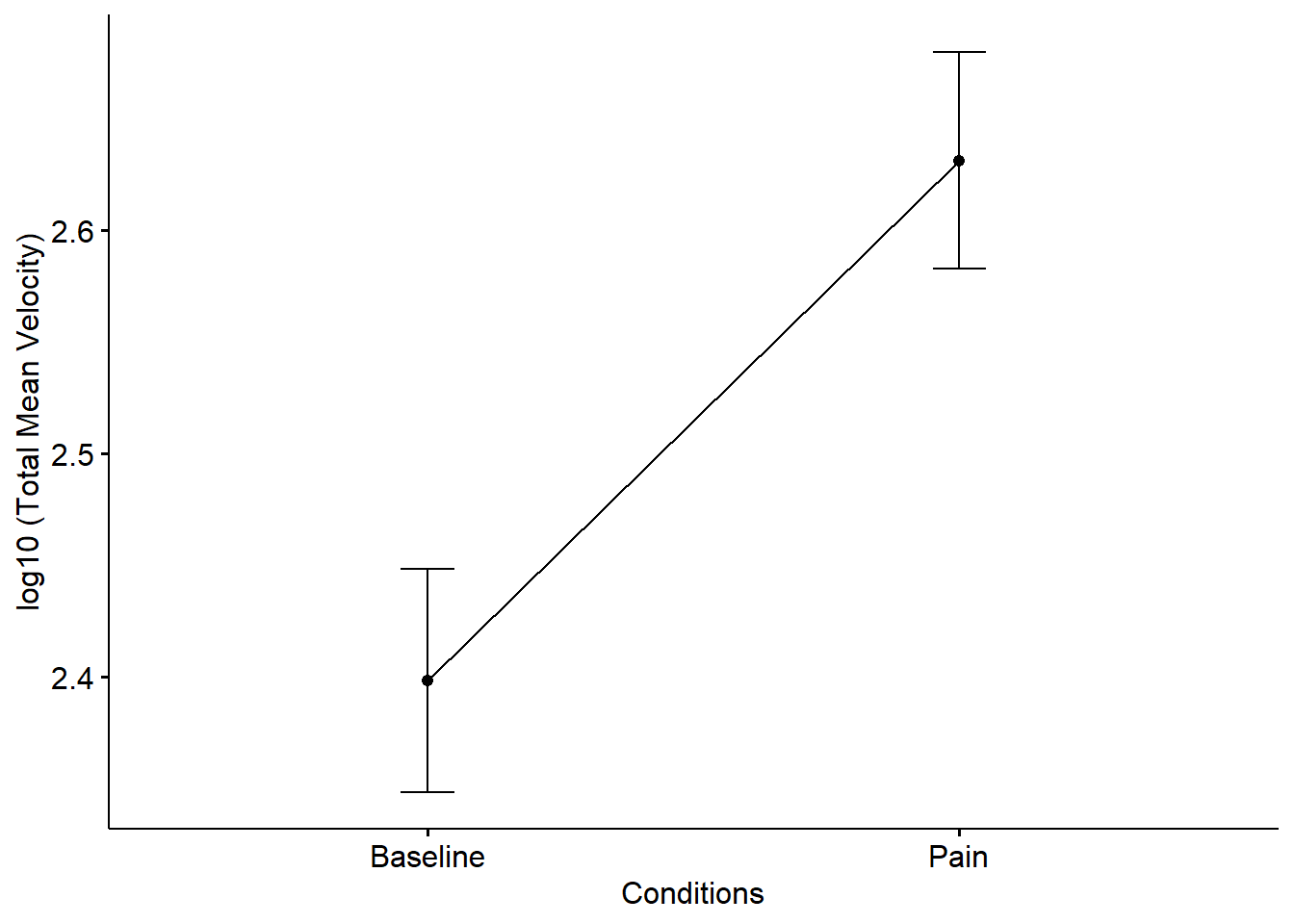


**Figure S1.** Log-transformed COP total mean velocity in the pain and baseline conditions. A significant increase in velocity was observed in the pain condition compared to baseline. Mean and standard deviation are displayed in the figure.

**
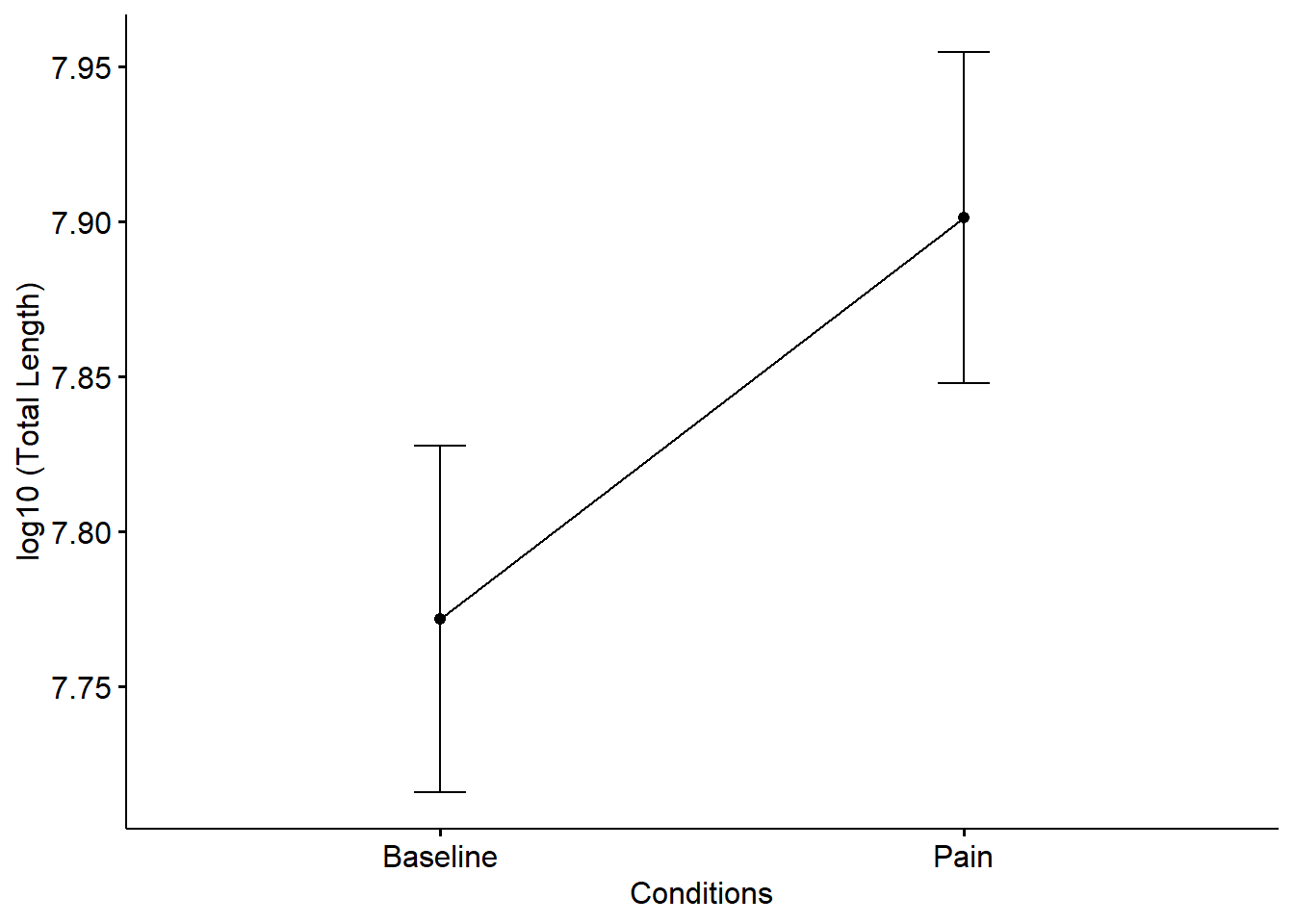
**

**Figure S2.** Log-transformed COP total length in the pain and baseline conditions. A significant increase in length was observed in the pain condition compared to baseline. Mean and standard deviation are displayed in the figure.

**Supplementary Table 1: Outlier exclusion by dependent variable and experiential structure**

| **Dependent Variable** | **Structure** | **N total** | **N excluded (extreme)** |
| --- | --- | --- | --- |
| **COP mean velocity** | Embodied Resonance | 25 | 1 |
| **COP mean velocity** | Marginal Embodied Resonance | 17 | 0 |
| **COP path length** | Embodied Resonance | 25 | 1 |
| **COP path length** | Marginal Embodied Resonance | 16 | 1 |
| **Heart rate (HR)** | Embodied Resonance | 23 | 3 |
| **Heart rate (HR)** | Marginal Embodied Resonance | 15 | 2 |
| **Phasic EDA** | Embodied Resonance | 21 | 5 |
| **Phasic EDA** | Marginal Embodied Resonance | 13 | 4 |

**Supplementary Table 1**: Number of excluded observations following outlier detection for each dependent variable and experiential structure. Outliers were identified separately for each structure *and condition* using an interquartile range–based criterion. Extreme values were defined as observations exceeding 3 × the interquartile range from the first or third quartile. For transparency, exclusions are reported here collapsed at the level of experiential structure. If a participant exhibited an extreme value in any condition (baseline or pain) for a given dependent variable, that participant was excluded from all statistical analyses and figures for that variable. All exclusions were applied prior to inferential analyses.

**Examples of Interview**

**Below are two examples of phenomenological interviews. The interviews were translated into English. “I” represents the interviewer and “P” represents the participant. In some interviews, the audio recording began after the description of the interview’s objective and instructions.**

**Example 1**

**I:** Okay. So, I’m going to ask you to connect with that experience, to evoke it, to recall it, to place yourself again in the moment of watching that video. Try to relive the sensations you may have experienced. And when you’re ready, please begin to describe what it was like for you to be standing on the platform, watching that specific video.

**P:** Well, I knew what was coming, that he was going to have an accident, that something was going to happen to him, but I never imagined that he would land upright, standing, and that, essentially, his entire skeletal area would be shattered, and that he probably wouldn’t be able to walk anymore. He tried to stand up, but he couldn’t.
So, what happens to me with that video is this sense of… how is it possible that we are capable of experiencing such a violent fall? I mean, it was obvious, it was clear, that he was going to crash.

**I:** Right. So, from the beginning, were you able to anticipate the fall?

**P:** Yes, it was clear that a serious accident was coming.

**I:** Okay. So you anticipate the fall, then the athlete jumps and falls.

**P:** He falls.

**I:** How was that moment for you, when he jumps and falls?

**P:** There was complete certainty that something was going to happen. But the strongest thing, from that part of the video, and I still feel it for him even now, was realizing that he was going to be seriously affected. Then I see that he is affected. What I didn’t know was where, whether it would be the knee, the head, the shoulders, the hips.
So that loss of certainty, combined with the feeling that he was going to be unable to walk, unable to stand, probably disabled for the rest of his life, that was very intense.

**I:** I see.

**P:** And then imagining what would come next. How severely affected he would be by the impact, and how he hadn’t measured the consequences or anticipated the problem that would result from the accident.

**I:** Right, so your impression was something like: how could he not anticipate the consequences?

**P:** Exactly.

**I:** And how was the moment when you saw that he was already injured?

**P:** Seeing him affected me deeply. I was struck by the fact that his hips were injured.

**I:** When he fell?

**P:** Yes.

**I:** And after that?

**P:** He stays lying there and tries to stand up, but he can’t.

**I:** Okay. Did something happen in your experience at that moment?

**P:** I imagined the pain he must have felt trying to stand up, because that whole area must have been completely dislocated. And the second thought was: what is he going to do now? What comes next for him? Trying to recover, and whether he will recover at all. I started thinking about all the tremendous consequences of that accident.

**I:** So, your experience had three moments: first, anticipating the fall,

**P:** Exactly.

**I:** Then the athlete falls and you notice that his hips are injured,

**P:** Yes.

**I:** And then he remains lying there and,

**P:** I begin to connect with the consequences.

**I:** With the consequences, right. So, how was your body during that first moment, when you anticipated the fall?

**P:** I was tense. I was in a state of readiness, prepared to receive the information coming from the jump.

**I:** Your whole body?

**P:** My whole body, yes. I was like a cat.

**I:** Like a cat, what do you mean by that?

**P:** A kind of bodily tension.

**I:** Okay.

**P:** Not slight, I think it was a lot of tension.

**I:** A lot of tension.

**P:** Yes.

**I:** Then the guy falls, boom!, and you focus on the hips.

**P:** Yes, and I feel it, I feel it myself.

**I:** I see.

**P:** It doesn’t hurt, but I feel it in my body, maybe right there where he is injured (points to hips).

**I:** Right.

**P:** And I think: that area is what allows movement.

**I:** Uh-huh.

**P:** The whole chain of leg movement, if that is damaged, there is no movement.

**I:** So you extend that perception to your own bodily chains.

**P:** Exactly. And I feel it.

**I:** And how would you describe that sensation?

**P:** Not pain, but something similar, like there was something there, in my hips.

**I:** And then?

**P:** Then I place myself next to him, like in a kind of battlefield…

**I:** Uh-huh.

**P:** On the ground, with him. I was watching him, but my sensation was to be next to him, talking to him, trying to encourage him, offering help.

**I:** I understand. Thank you very much.

**Example 2**

**E:** So, Mrs. [Name], let’s focus on your experience during the video you selected. Could you describe how you lived it, how you experienced it?

**P:** Yes, it’s a boy climbing between two walls, and he falls.

**E:** Let’s take a few seconds for you to relive and recall what you experienced with that video.

**P:** What happened is that with negative things, I tend to detach. So, I watched it and thought, “How reckless, how stupid,” but I don’t get emotionally involved with these things. It’s not like… I know people who see something like that and start crying.

**E:** I understand.

**P:** For me, it leaves me indifferent. Apart from thinking “how reckless,” I don’t get involved any further.

**E:** Uh-huh.

**P:** I can’t feel any emotion, any sentiment beyond thinking: “How stupid, what were they thinking to do something like that?”

**E:** I see.

**P:** These kinds of things don’t generate anything in me; they don’t make me feel any emotion or sensation. From the first moment to the end, I just think it’s stupid.

**E:** I understand.

**P:** I don’t understand how people do such brutal things.

**E:** I see. If we recap a little, in this video, the person is climbing between the two walls, reaches the top, tries to climb the building, fails, and falls.

**P:** Yes, and he falls partly because of how he’s holding on. I was paying attention to that as well.

**E:** I understand. And did your experience change across these three moments, or how did it unfold?

**P:** It was consistent the whole time; it remained steady throughout the video. There was no change or increase in intensity.

**E:** How did your body feel, for example, watching the athlete suffer?

**P:** Just observing. I was like a spectator, not involved—just like watching any other video. I was completely outside of the screen.

**E:** Did you feel any motivation while watching this young man in the video?

**P:** Motivation to do something? Not at all. I had no motivation whatsoever.

**E:** I see.

**P:** Well, throughout the video, I had this thought, this evaluation, that it was pure recklessness. Yes, I thought the whole time that it was stupid, reflecting on how foolish people are to do these things.

**E:** Uh-huh.

**P:** There are people who are simply very foolish to do this.

**E:** I understand.

**P:** It was like watching something completely unrelated to me; I was only thinking about these young people doing reckless things.

**E:** I understand that you had those thoughts. Thank you very much for your description.

**P:** You’re welcome.
