# Supplementary material for: When the body resonates with the pain of the other: Empathy Bodyssence in Parkinson’s disease
Source: Neurosci Conscious. 2026 Apr 7;2026(1):niag010. doi: 10.1093/nc/niag010 (PMC13064857; doi:10.1093/nc/niag010)
Supplement: niag010_Supplementary_materials [file niag010_supplementary_materials.pdf]

CODEBOOK

When the Body Resonates with the Pain of the Other: Empathy Bodyssence in Parkinson's Disease

María del Carmen Tejada, Antonia Zepeda, Alejandro Troncoso, Anaís Aluicio, Rebecca Todd, David Martínez Pernía

The following codebook presents and describes the four main themes that constituted the lived experiences of empathy for pain in patients with Parkinson's disease. The four emerging main themes were: "bodily resonance", "motivation", "sense of ownership" and "internal dialogue". Additionally, the subthemes comprising each main theme are delineated.

Building on Mihas's (2019) contributions to codebook development, each main theme and subtheme was documented using four core elements: a description, its relevance, an illustrative example, and a reflective component. The description explains how the code was applied during the analytic process, while the relevance clarifies its function within the phenomenon under study. The example consists of a verbatim excerpt that concretely exemplifies the code. The reflective component addresses how the code may have emerged, evolved, or been refined across different stages of the analysis. In addition, each main theme is preceded by a code diagram that visually depicts the relationships among main themes, subthemes, and subcategories identified in the analysis.

1) MAIN THEME: BODILY RESONANCE

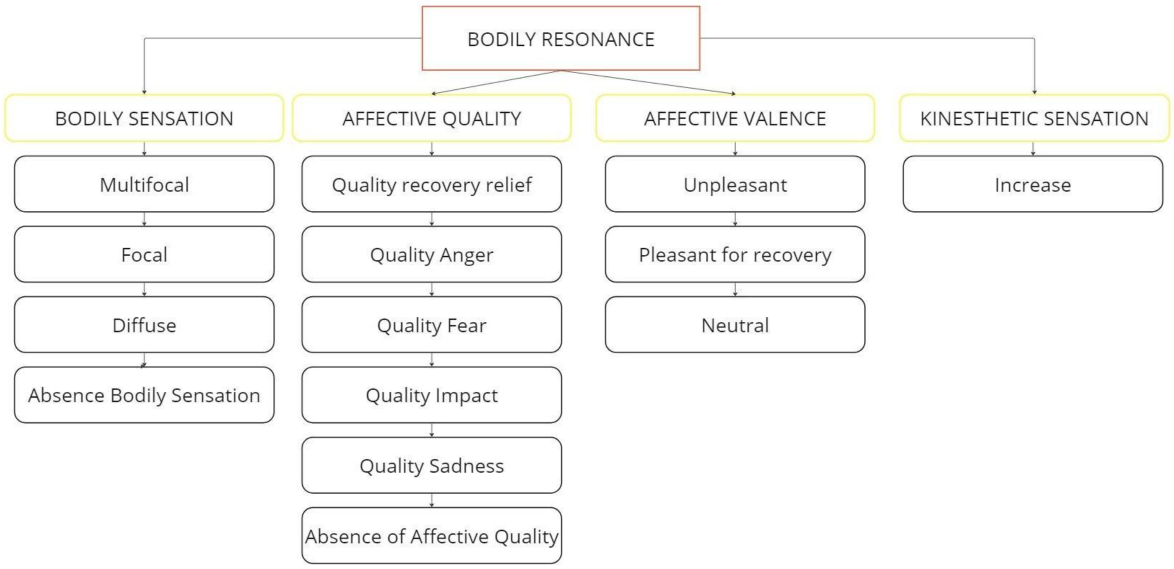

Description: Reflects that participants experienced a form of engagement with the suffering of the other, lived through bodily and emotional sensations that arise synchronously with the athlete's behavior. As participants observe, their bodily experiences resonate with the athlete's

movements, manifesting in a spectrum of bodily sensations, emotional responses, and kinesthetic experiences.

Importance: The relevance of this term is fundamental, as it clarifies the role of corporeality in the subjective experience of empathy for pain. It is within this central theme that the forms of engagement can be observed through the presence or absence of participants' bodily and affective sensations while witnessing the athlete's experience.

Example:

[Referring to the moment of the fall of the athlete]: "P: Just before the fall I felt tension [...] with the fall, the body becomes more tense [...] when he falls, the tension rises, I saw the impact and tensed my body, I felt tense all over [...] I didn't like the fall, it was unpleasant..." (P 17)

Reflection: This central category has been previously utilized by this research group in other publications on empathy for pain, making it one of the most relevant analysis categories since the initial stage of phenomenological analysis.

1.2) SUBTHEME: Bodily Sensation

Description: Refers to the perception of the body's internal states when coming into contact with the suffering of the other. Within this perception, participants experience a range of muscular and visceral sensations, which may be localized in specific areas (focal) or distributed across multiple regions simultaneously (multifocal). The specific areas affected include the upper and lower extremities, neck, trunk, face, chest, and abdomen. Some participants also described muscular sensations in vague or very broad (diffuse) areas of the body. In contrast, some participants experienced an absence of bodily sensations when observing the athlete's suffering, perceiving a continuity in their bodily experience without undergoing changes in response to the athlete's suffering.

Examples:

[referring to the moment of the fall of the athlete]: "Just before the fall I felt tension [...] with the fall, the body becomes more tense [...] when he falls, the tension rises, I saw the impact and tensed my body, I felt tense all over [...] I didn't like the fall, it was unpleasant..." (P 17)

[referring to the moment the athlete hit the ground] "I felt pity, anguish, I thought, poor guy [...] I felt tense, clenching my stomach and holding it in, tight, tense..." (P 25)

[Referring in general to the scene]: "P: I was worried, knowing that if he falls, he will die, and that is kind of sad [...] I thought the one who was climbing well, I thought he was almost there, and it made me sad that just when he was about to reach the top, he ends up with nothing. I: did you feel any sensation in your body, a change, anything? P: No, no, no, nothing [...] there was no shock, no tension, nothing " (P1)

[Referring to the moment of the fall]: " I: Did you feel any sensation in your body while you were watching the fall? P: No, I didn't feel anything [...] I was just watching an image, the image shown on the television, just like that. I: And emotionally, while watching that image? P: No,

nothing, I didn't feel anything. I: And during the entire time the scene lasted? P: No, nothing. " (P12)

Reflection: This subtheme emerges in the initial phase of the analysis process, and its early appearance is attributed to the significance of corporeal-affective responses in the participants' empathetic experience within the context.

1.3) SUBTHEME: Affective Quality

Description: Refers to the specific types of emotional responses experienced by participants in relation to the athlete's actions, including emotions such as anger, fear, sadness, and also the absence of emotional responses.

Importance: This subtheme holds significant relevance, as it integrates the affective dimension in relation to bodily sensations. It details the type of emotion participants experience at different moments in the video.

Example:

[referring to the moment just before the fall] "P: I curled up entirely because I saw that, that it was going to happen and that he couldn't stop the accident that it was. So, I felt nervous, I felt that I moved a lot and it was like I was expecting something fatal, something very bad, very bad [...] I felt something here, in my chest, I had to take a deep breath, I: What emotion are you describing? P: Sadness for what is going to happen" (P50)

[Referring to the moment of impact of the athlete with the ground]: >No, it didn't cause me anything< (P 24)

Reflection: This subtheme emerges in the initial phase of the analysis process, and its early appearance is attributed to the significance of emotional responses in the experience of witnessing another's suffering.

1.4) SUBTHEME: Affective Valence

Description: It refers to the unpleasant, pleasurable for recovery or neutral character of the emotional responses experienced by the participants in response to the suffering of the other. During the anticipation and observation of the athlete's fall, most participants experience an unpleasant affective valence, feeling emotions such as anger, fear, and sadness. However, toward the end of the scene, upon confirmation that the athlete is okay, some participants feel a pleasant affective valence, described as a sense of relief and emotional recovery to a state similar to that prior to the fall. In contrast, some participants experience a neutral affective valence, experiencing no emotional change, regardless of the observed distress.

Importance: It is a crucial dimension for understanding the perceived emotional nature of the stimulus, providing a context to comprehend affective qualities and thus enabling a profound understanding of them.

Example:

[referring to the moment of the fall]: "P: you feel unpleasant, with sorrow [...] worry because you are going to suffer, maybe for how long" (P14)

[referring to the moment after the fall]: "P:well there (.) there is a kind of relief because the man is not destroyed below and fortunately he is saved and what I feel there I feel joy, I feel the joy of relief [...] as if this part between the chest and the throat, up here, it relaxes" (P46).

[referring to the moment of the fall] "I: how did you feel when you saw it, the accident? P: >no, it didn't cause me anything [...] it caused me, it caused me::: like a general thing, like seeing pictures [...] neutral"

Reflection: Similar to the previous subtheme, this one emerges in the initial phase of the analysis process, driven by the need to comprehensively account for emotional responses, including their valence and quality.

1.5) SUBTHEME: Kinesthetic Sensation

Description: This subtheme refers to the pre-reflexive body movements that participants experienced in response to the suffering of the other. Some participants felt a sensation of movement when they saw the athlete fall, experiencing a change in their sense of postural balance or a slight forward lean when the athlete fell.

Importance: It is relevant to characterize the bodily experience that arises in participants when they see another suffer. Specifically, their information refers to the pre-reflexive movements that the participants experienced in coordination with the actions of the athletes.

Example:

[Referring to the moment before the fall] "Um::: I lost my balance or well, I didn't lose it, I moved a little... to help him, to assist him" (P 35)

[Referring to the moment of impact of the athlete with the ground]: "Like when he jumped and fell, made contact with the snow, and then started descending. And I noticed a slight imbalance forward" (P 45)

Reflection: Arises in the initial phase of the analysis to capture sensations of movement perceived by the participant in response to the scene.

2) MAIN THEME: MOTIVATION

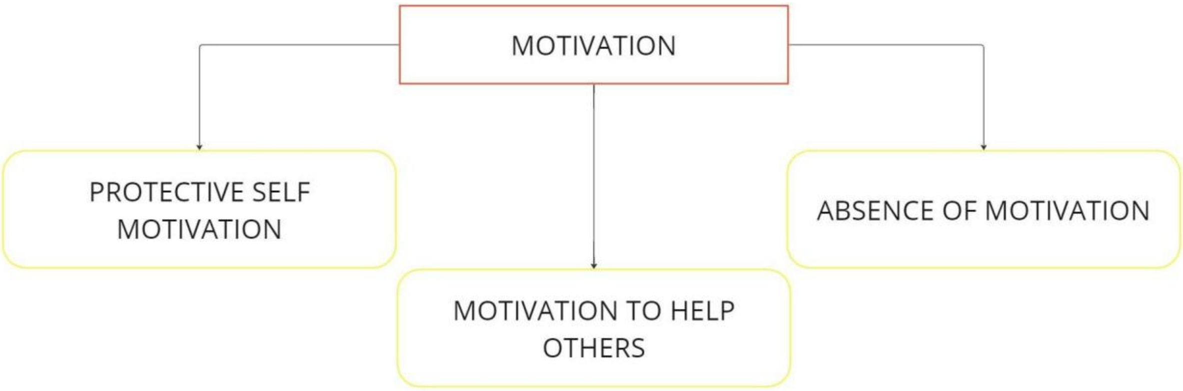

Description: Participants described a pre-reflective impulse toward action or non-action while observing the athlete. They experienced spontaneous reactions to different moments of the scene, reflecting a variety of goal-oriented tendencies that emerge when witnessing another person's suffering. These motivations revealed three distinct kinesthetic tendencies among participants: an impulse to help the other, a self-protective impulse, and the absence of motivation.

Importance: Motivation stands out as a category that adds nuance to the empathic experience of pain in Parkinson's, accounting for pre-reflective impulses emanating in each participant according to their experience.

Example:

[Referring to the moment of the fall]: P: While I was watching the guy while he was climbing and then when he goes when he was climbing and when he lets go and falls I wanted to put something to save him or help him so that he wouldn't get hit [...] I could see myself there with him (P23).

Reflection: This central theme emerges in the initial phase of the analysis, initially composed of two impulses, the self-protective impulse, and the impulse to help others. In the second phase of the analysis, the absence of motivation was included, broadening the range of goal-directed responses.

2.1) SUBTHEME: Protective Self Motivation

Description: It refers to the experience of some participants who expressed a desire to stop watching the athlete or to turn off the scene, while simultaneously experiencing a self-protective impulse directed toward themselves, manifested as a bodily urge to cover their face.

Importance: This subtheme shows how self-protective motivation is intertwined with participants' bodily tendencies.

Example:

[Referring to the moment of the fall]: "I: Any other sensation you had while watching that? P: Wanting to escape from it, wanting to ignore it, not wanting to know, wanting to escape from it, wanting to ignore it [...] here I watched the scene because I had to, but I try not to watch or change, I try to avoid jumps" (P 4).

Reflection: This subtheme emerges in the initial phase of the analysis due to the early appearance of this impulse in the participants' experiences.

2.2) SUBTHEME: Motivation to help others

Description: It refers to the experience of another group of participants who experienced a motivation to help the athlete. While observing different moments of the scene, they felt a pre-reflective kinesthetic impulse to intervene and assist. They perceived an intense desire to alleviate the athlete's suffering, which manifested in action tendencies such as warning the athlete of danger, alleviating their pain, or seeking medical help.

Importance: This subtheme shows how help others' motivation is intertwined with participants' bodily tendencies.

Example:

[Referring to the moment before and after the fall]: "I: How was your experience when you saw the athlete climbing? P: I was focused on trying to help, feeling like I wanted to tell him that it was wrong, that the sport was too risky for him. I: How was your feeling when the accident happened? P: Intense urge to tell him no, to not do it! " (P29)

Reflection: Similar to the previous subtheme, this is part of the initial phase of the analysis, as the prosocial response of helping others manifested early in the participants' experiences.

2.3) SUBTHEME: Absence of Motivation

Description: In contrast to those participants who experienced a pre-reflective impulse to protect or help the other, there were also participants who felt a complete absence of pre-reflective impulses related to the athlete's suffering.

Importance: This subtheme accounts for the experience of a group of participants who lack any pre-reflective impulse when watching the video, providing details on how that part of the experience is lived, characterizing it.

Example:

[Referring to the moment of the fall]: " I:Did you have any sensation watching the athlete go up? P: just watching [...] I was just watching as a spectator without getting involved. I: did you feel like doing anything? [...] P: deep down I saw it as if it was a scene [...] whatever I did would be useless, I didn't feel like doing anything. " (P40)

Reflection: This subtheme emerges in the second phase of the analysis due to the need to identify those individuals who did not feel any motivation when watching the athlete fall.

3) MAIN THEME: INTERNAL DIALOG

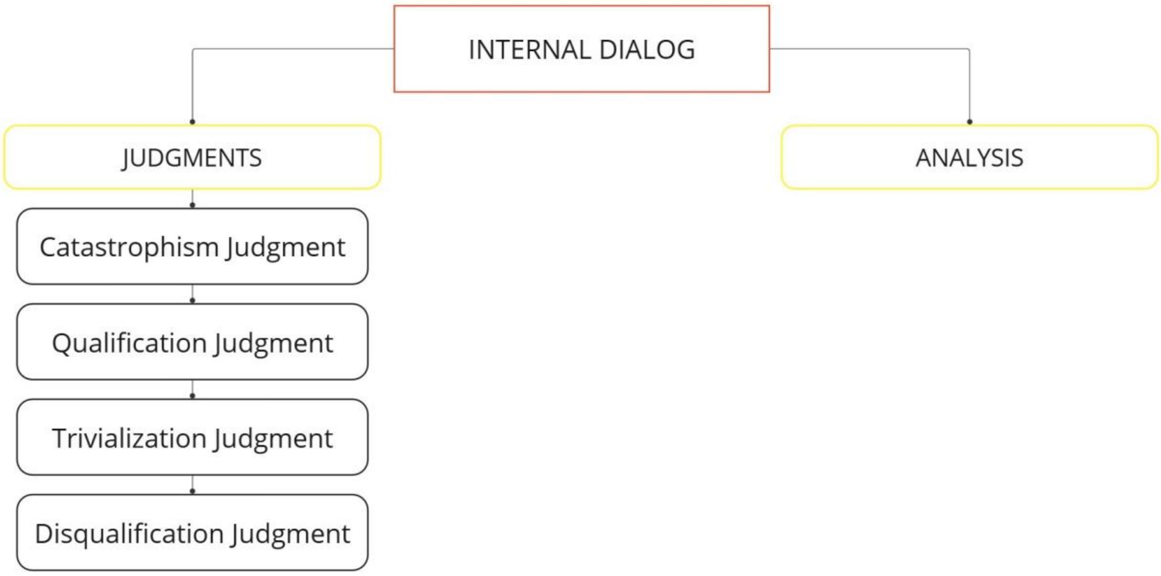

Description: Participants have an internal dialogue manifested as a constant flow of interwoven thoughts and reflections that unfold as they witness the athlete's suffering. This internal dialogue takes two distinct forms: making judgments about what they are witnessing and reflecting in detail on the technical aspects of the fall.

Importance: This sub-theme allows us to understand the type of internal dialogue that the participants had with themselves and how this dialogue was intertwined with the different temporal moments of the experience.

Example:

[referring to the moment of the fall]: P: while I was watching the guy while he was climbing and then when he goes when he was climbing and when he lets go and falls I wanted to put something to save him or help him so that he wouldn't get hit [...] I could see myself there with him (P23).

Reflection: This central theme emerges in the initial phase of analysis. While some subthemes were added in other analysis processes, it emerges as a way to portray the cognitive component of empathy.

3.1) SUBTHEME: Judgments

Description: Some participants, when viewing the scene, make judgments about different elements, some are involved in a whirlwind of thoughts projecting the possible consequences of the action they are witnessing, manifesting a judgment of catastrophism that leads them to imagine the worst possible scenarios. Others, meanwhile, embark on a subjective evaluation of the person or sporting activity they are witnessing, using a rating judgment to weigh its

quality or appropriateness. In addition, there are participants who began to evaluate the athlete's accident from a perspective of trivialization, perceiving the event as banal or ordinary, without taking into account the seriousness or suffering of the athlete. Finally, the internal dialogue of some participants had to do with criticizing the person or the sport activity.

Importance: Provides insight into the evaluations and opinions of participants regarding the action observed in the video.

Example:

[Referring to the moment of the impact of the athlete with the ground]: "P: because (thinking) that he/she was going to be very fractured (could) cause death " (P 28).

[Referring to the moment before the fall] "P: that it was irresponsible, irresponsible let's say in the aspect that one also has to evaluate many things..." (P 9)

[Referring to the moment after the fall]: "P: I found that he was an idiot haha" (P 14)

[Referring to the moment of the impact of the athlete with the ground]: "P: not to pay attention to it because eh as I say ( ) it's just silly" (P 21)

Reflection: This subtheme is part of the initial phase of the analysis, initially composed of catastrophism, qualification, and disqualification judgments. In the second phase of the analysis, trivialization judgment was added, concluding the subtheme.

3.3) SUBTHEME: Analysis

Description: Some participants experienced the emergence of an analytical internal dialogue. During this process, they perceived themselves as maintaining a detailed analysis of the technical aspects of the fall, meticulously describing and reviewing the actions and elements that make up specific moments of the scene.

Importance: This subtheme provides clues about the elements of the scene that participants focus on and how those elements are integrated into their internal dialogue during the experience.

Example:

[Referring to the moment of the impact of the athlete with the ground]: "P: he is already hanging from the mountain, and there is a jump and there he loses concentration, because he loses concentration and falls, he falls in a very bad way, because he loses concentration, he loses track of what he is doing..." (P 6)

Reflection: The analysis subtheme emerges in the initial phase of the analysis without subsequent transformations or modifications.

4) MAIN THEME: SENSE OF OWNERSHIP

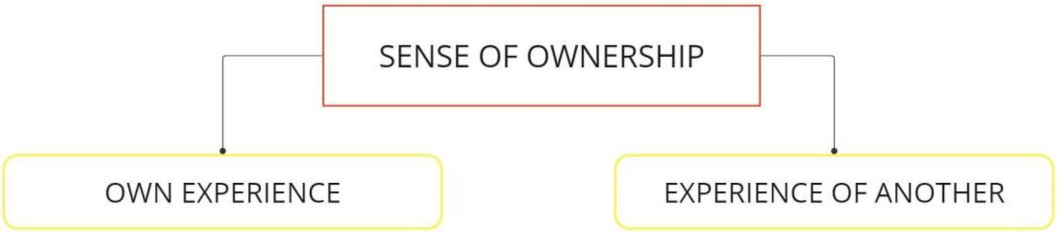

Description: The sense of ownership intertwines with the intimate perspective a participant assumes when witnessing another person's suffering, encompassing how and where they direct their attention, emotions, and corporeal sensations. The sense of ownership describes how the participant internally positions and connects when witnessing another's pain.

Consider a self-centered sense of ownership and an other-centered sense of ownership.
Importance: It allows us to show the different intimate perspectives that participants adopt in response to the suffering of another, offering nuances about the different ways in which participants focus and direct their attention, emotions and bodily sensations during the observation of the scene.

Example:

[Referring to the moment after the fall where the athlete remains on the ground]: "P: No, no, there sadness, concern, THINKING ABOUT ONESELF, thinking about the family, thinking that if it happened to me or someone from my, known people, it would be TERRIBLE" (P 25)

[Referring to the moment before the fall]: I: And how did you feel when you were watching this guy falling? P: I felt worried and I kept staring, watching how the fall was going and thinking that his lifeline would not be cut [...] I was watching everything [...] always thinking that the guy would make it to the bottom, that the rope would work [...] I could see myself putting a mat there, so he wouldn't fall.

Reflection: The genesis of this central theme originates in the second period of analysis, with the purpose of providing a more precise and integrative description of the elements related to the intimate perspective of the participant in relation to the suffering of the other.

4.1) SUBTHEME: Own Experience

Description: It is characterized by participants experiencing the athlete's situation as directly affecting them. Participants feel unpleasant emotions intertwined with physical sensations of discomfort and yet maintain attention on themselves, connected during the viewing of the scene to the personal distress produced by the athlete's suffering.

Importance: The importance of this sub-theme lies in accounting for experiences in which participants witnessing the suffering of another, direct their focus of experience to their own discomfort. It provides a better understanding of the structures of experience in which this self-centered perspective exists.

Example:

[Referring to the moment of the fall]: "I: How did you feel on an emotional level? P: it hurt me [...] yes, like how could it be to do such a strong thing? I don't know, I couldn't, no no no, if I saw someone from my family, from my people doing something like that and that happened to them it would hurt me a lot, I wouldn't have conformity." (P15)

Reflection: This subtheme emerges in the second phase of analysis and does not undergo modifications once defined.

4.2) SUBTHEME: Experience of Another

Description: Some participants experience a sense of ownership centered on the athlete's experience. During exposure to the scene, these participants feel a deep affective and corporeal response when watching the athlete suffer, focusing their attention and bodily dimension on the athlete's suffering. They maintain a perspective primarily centered on what the athlete is experiencing, sustaining moment-to-moment attention and connection to the athlete's situation.

Importance: This sub-theme provides insight into the experience of a group of people who, in witnessing the suffering of another, consistently direct their intimate perspective toward that other. It helps to understand the structures of a more empathic experience, in which the suffering of the other is the focus of attention.

Example:

[Referring to the moment before and after the fall]: "P: I was certain that something was going to happen. But the strongest thing, from that part of the scene, that I still feel for him, is to have realized that he was going to be (affected) [...] I was watching him [...], my feeling is to be next to him, talking to him, trying to encourage him. " (P20)

Reflexion: In the same line as the previous subtheme, this emerges in the second phase of analysis. Initially, this sub theme consisted of codes that alluded to the attentional component. In the second phase of analysis, it was refined and consolidated.

REFERENCES

Mihas, P., & Odum Institute. (2019). *Learn to build a codebook for a generic qualitative study*. SAGE Publications, Limite
